# Posterior cingulate cortex reveals an expression profile of resilience in cognitively intact elders

**DOI:** 10.1093/braincomms/fcac162

**Published:** 2022-06-21

**Authors:** Christy M Kelley, Stephen D Ginsberg, Winnie S Liang, Scott E Counts, Elliott J Mufson

**Affiliations:** Department of Translational Neuroscience, Barrow Neurological Institute, St. Joseph’s Hospital and Medical Center, Phoenix, AZ 85013, USA; Department of Neurology, Barrow Neurological Institute, St. Joseph’s Hospital and Medical Center, Phoenix, AZ 85013, USA; Center for Dementia Research, Nathan Kline Institute, Orangeburg, NY 10962, USA; Department of Psychiatry, New York University Grossman School of Medicine, New York, NY 10016, USA; Department of Neuroscience & Physiology, New York University Grossman School of Medicine, New York, NY 10016, USA; NYU Neuroscience Institute, New York University Grossman School of Medicine, New York, NY 10016, USA; Translational Genomics Research Institute, Phoenix, AZ 85004, USA; Department of Translational Neuroscience, Michigan State University College of Human Medicine, Grand Rapids, MI 49503, USA; Department of Family Medicine, Michigan State University College of Human Medicine, Grand Rapids, MI 49503, USA; Department of Translational Neuroscience, Barrow Neurological Institute, St. Joseph’s Hospital and Medical Center, Phoenix, AZ 85013, USA; Department of Neurology, Barrow Neurological Institute, St. Joseph’s Hospital and Medical Center, Phoenix, AZ 85013, USA

**Keywords:** aging, cognition, human, posterior cingulate cortex, RNA-Seq

## Abstract

The posterior cingulate cortex, a key hub of the default mode network, underlies autobiographical memory retrieval and displays hypometabolic changes early in Alzheimer disease. To obtain an unbiased understanding of the molecular pathobiology of the aged posterior cingulate cortex, we performed RNA sequencing (RNA-seq) on tissue obtained from 26 participants of the Rush Religious Orders Study (11 males/15 females; aged 76–96 years) with a pre-mortem clinical diagnosis of no cognitive impairment and post-mortem neurofibrillary tangle Braak Stages I/II, III, and IV. Transcriptomic data were gathered using next-generation sequencing of RNA extracted from posterior cingulate cortex generating an average of 60 million paired reads per subject. Normalized expression of RNA-seq data was calculated using a global gene annotation and a microRNA profile. Differential expression (DESeq2, edgeR) using Braak staging as the comparison structure isolated genes for dimensional scaling, associative network building and functional clustering. Curated genes were correlated with the Mini-Mental State Examination and semantic, working and episodic memory, visuospatial ability, and a composite Global Cognitive Score. Regulatory mechanisms were determined by co-expression networks with microRNAs and an overlap of transcription factor binding sites. Analysis revealed 750 genes and 12 microRNAs significantly differentially expressed between Braak Stages I/II and III/IV and an associated six groups of transcription factor binding sites. Inputting significantly different gene/network data into a functional annotation clustering model revealed elevated presynaptic, postsynaptic and ATP-related expression in Braak Stages III and IV compared with Stages I/II, suggesting these pathways are integral for cognitive resilience seen in unimpaired elderly subjects. Principal component analysis and Kruskal–Wallis testing did not associate Braak stage with cognitive function. However, Spearman correlations between genes and cognitive test scores followed by network analysis revealed upregulation of classes of synaptic genes positively associated with performance on the visuospatial perceptual orientation domain. Upregulation of key synaptic genes suggests a role for these transcripts and associated synaptic pathways in cognitive resilience seen in elders despite Alzheimer disease pathology and dementia.

## Teaser

Kelley *et al.* report RNA-Seq derived gene expression in the posterior cingulate cortex of aged, non-demented humans staged post-mortem for neurofibrillary tangle pathology. The authors observed enrichment in synaptic pathways for Braak Stages III and IV compared with Stages I/II, highlighting a possible mechanism for cognitive resilience.

## Graphical Abstract

**Graphical abstract:**
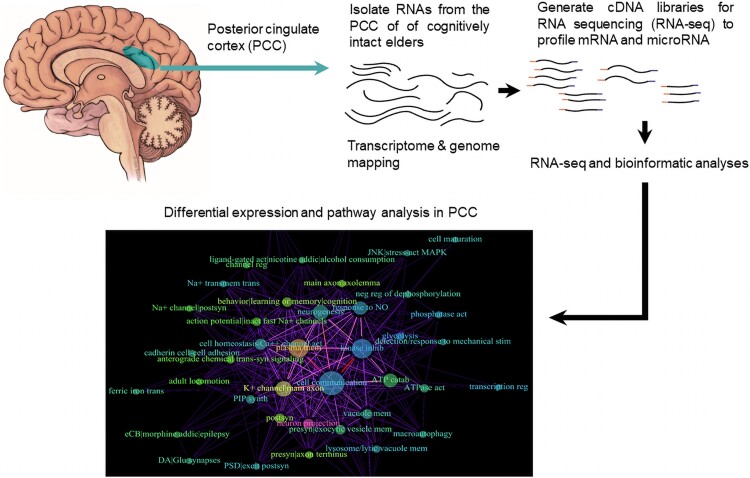

## Introduction

Alzheimer disease is a major public health issue resulting in significant societal and economic burden.^1^ Alzheimer disease is considered a spectrum disorder,^2–4^ characterized clinically with declining memory, executive function and an inability to perform activities of daily living.^5,6^ Neuropathologically, it is characterized by neurofibrillary tangles (NFTs) containing hyperphosphorylated tau, insoluble amyloid plaques, increased production of amyloid-beta peptide (Aβ) species, neuroinflammation and synaptic loss.^7–9^ Although NFTs are associated with both Alzheimer disease progression and cognitive decline,^10–12^ they are not absolute predictors of dementia. At least 15% of adults display NFTs in the medial temporal lobe (MTL) memory circuit characterized as Braak Stage I–II showing an age-associated elevation of tau pathology in cross-sectional health populations.^13,14^ Interestingly, elders with a pre-mortem clinical diagnosis of no cognitive impairment (NCI) met criteria for Braak NFT stages ranging from I–VI ^15–17^ suggesting NFT pathology is not necessary for cognitive impairment. Identifying the molecular pathogenesis underlying brain resilience to cognitive decline despite varying stages of NFT pathology will provide new avenues for intervention to delay the onset of Alzheimer disease, an unmet need and a priority for the National Institute on Aging (NIA).^18^

**Figure 1:**
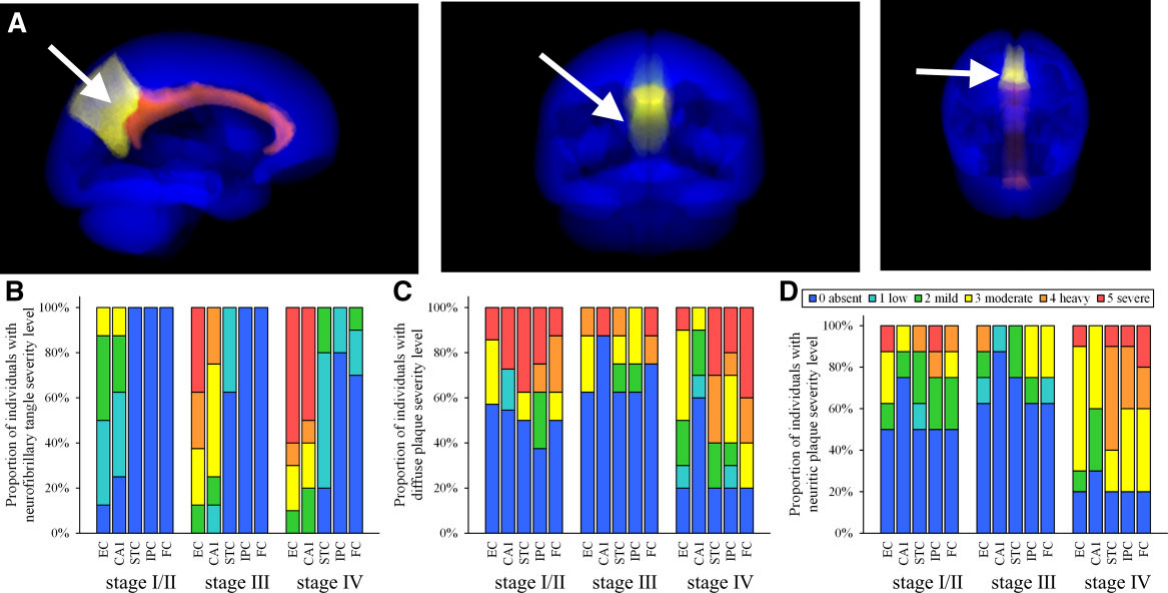
**Location of PCC and distribution of NFTs and amyloid pathology in RROS cases.** (**A**) Images generated in Image J using the SRI24 human brain atlas^135^ indicating location of the PCC (arrow) ventral to the precuneus (yellow) and dorsal to the corpus callosum (orange) shown in the sagittal, coronal and horizontal planes. (**B**) Bar graphs showing cortical region and severity of NFT pathology across Braak stages in NCI cases used for PCC expression profiling. NFT pathology was less in the entorhinal cortex (EC) and CA1 sector of the hippocampus in Braak Stages I/II (*n* = 8) and increased in Stages III (*n* = 8) and IV (*n* = 10). The superior temporal cortex (STC), inferior parietal cortex (IPC), and frontal cortex (FC) were virtually devoid of NFTs in Braak Stage I/II and III, contrasting with Stage IV. C, D. Bar graphs depicting diffuse Aβ (**C**) and neuritic plaque (**D**) regional distribution varied across Braak stages. At least 50% of Stage I/II and III cases displayed no or low plaque load, whereas Stage IV varied from absent to severe diffuse and neuritic plaques across the brain regions examined.

Although the MTL is an early site for NFTs,^19–21^ the posterior cingulate cortex (PCC), a hub of the cortical default mode network^22^ (DMN, Fig 1A), that plays a role in autobiographical memory retrieval, attention, salience and emotional context,^23,24^ displays metabolic dysregulation during the onset of Alzheimer disease.^25–28^ Neuroimaging studies indicate the DMN monitors the external and/or internal environment.^29–31^ The PCC is dysregulated at resting state and during attention-demanding tasks in individuals with mild cognitive impairment (MCI) and Alzheimer disease.^32,33^ Unlike other DMN hubs (e.g. precuneus, prefrontal cortex),^34^ there are no standalone clinical molecular transcriptomic studies of the PCC from elderly people with a pre-mortem clinical diagnosis of NCI and a post-mortem Braak stage of I–IV, which may include a population resilient to the pathogenesis of Alzheimer disease. The lack of PCC transcriptomic information in elders with NCI, but with NFT pathology, impedes discovery science for therapeutics and understanding mechanisms underlying cognitive reserve/resilience that is not possible to model in preclinical animal and cellular preparations.

We performed high-throughput RNA sequencing (RNA-Seq), with subsequent specialized bioinformatic inquiry to assess genes and microRNAs (miRNAs) in association with clinical pathological variables using post-mortem PCC tissue obtained from elderly subjects that came to autopsy with a pre-mortem clinical diagnosis of NCI and received a post-mortem neuropathological Braak score of I–IV from the Rush Religious Order Study (RROS).^35,36^ The goal was to identify a transcriptomic baseline profile of the PCC in healthy aged individuals without cognitive impairment but with varying stages of NFT pathology to generate a putative molecular fingerprint of resilience within this key hub of the DMN.

### Materials and methods

The study cohort (*n* = 26) consisted of retired clergy with no signs of dementia at enrolment in the RROS, a longitudinal clinical pathological study.^35,37^ Cognitive testing was performed annually during life. Post-mortem brains were examined for neuropathologic features of Alzheimer disease and related disorders.^38^ Exclusion criteria included Lewy body dementia, Parkinson disease, hippocampal sclerosis, vascular disease and large strokes.^35,37,39,40^ Apolipoprotein E (*APOE*) genotyping was performed as previously reported^35,37,39,40^ and confirmed by RNA-Seq to identify nonsynonymous polymorphisms encoding base substitutions at amino acid positions 112 and 158.^41^

#### Clinical and neuropathological evaluations

Briefly, RROS testing included the Mini-Mental State Examination (MMSE)^42^ and a global cognitive score (GCS) compiled from a battery of 19 cognitive tests, which contribute to a cognitive domain score.^35,37^ Neuropathological diagnosis was based on Braak NFT staging, NIA-Reagan criteria and the Consortium to Establish a Registry for Alzheimer’s disease (CERAD).^43–45^ In addition, brain slabs containing the PCC were immersion fixed in 4% paraformaldehyde, cryoprotected, cut into 40 µm thick sections and two sections from each case were immunostained with an antibody against the amyloid precursor protein (APP) and Aβ (6E10, 1:400 dilution) and tau (AT8, 1:250 dilution) as previously reported.^46,47^ PCC 6E10 and AT8 loads were determined using a semi-quantitative score ranging from no 6E10-positive amyloid plaques and no AT8-positive NFTs, neurites or neuropil threads (0) to mild-to-moderate (2–3) to moderate-to-severe (4–5).

#### Preparation of tissue and RNA-Seq

PCC was excised using fiduciary landmarks^48,49^ and stored at -80 °C until processing at the Collaborative Sequencing Center (Translational Genomics Research Institute, Phoenix, AZ). Total RNA from frozen slabs was extracted (mirVana; Ambion, TX) with enrichment for small RNAs, enabling assessment of mRNAs and non-coding RNAs (ncRNAs) including miRNAs.^50,51^ Tapestation (DV200; Agilent, Santa Clara, CA) values ranged from 67.12% to 91.58%. RNA-Seq libraries were prepared using 500 ng of total RNA (TruSeq Stranded RNA Kit; Illumina, CA), ligated with xGen Dual-index UMI adapters (Integrated DNA Technologies, Coralville, IA) and enriched using eight PCR cycles. Libraries were paired-end sequenced (HiSeq4000, Illumina) for 80 base-pair (bp) reads.

#### Read processing

FastQ files were merged for paired ends before quality filtering and trimming using Fast Read Adjustment of Short reads (FLASH-1.2.11, minimum overlap 10 bases, maximum overlap 80 bases, mismatch allowed 1 in 4).^52^ Reads were trimmed (sliding window of 3 bases with an average quality ≥ 32), quality filtered (average trimmed read quality ≥ 30) and size-selected (≥ 50 bases) using Trimmomatic (0.32)^53^ resulting in three files per subject converted to fasta: single reads consisting of merged paired-end and R1 or R2 reads without a pair, R1 reads with a pair and R2 reads with a pair (see Supplementary Methods for details). The latter were collapsed into one paired reads file. The resulting two files (paired and unpaired) were mapped to *Homo sapiens* genome Genome Reference Consortium Human Build 38 patch release 13 (GRCh38.p13, hg38, assembly GCF_000001405.39), retrieved March 2020 (chr1-24, M), in Geneious using a custom annotation-span preference algorithm (v.9.0.1; Biomatters, Inc., CA). This involved a 13-mer index length (reads) and 18-mer word length (genome) and allowed for paired overlaps and gaps in reads as well as intron spanning. The hg38 genome was annotated using feature files for NCBI RefSeq, miRBase, LINC, and SNORD/miRNA. After mapping to somatic chromosomes 1-22 and X (NC_000001-NC_000023) and mitochondrion (NC_000025), unused reads were mapped to the Y chromosome (NC_000024) with no chromosome masking.^54,55^ Alignment files were exported and raw counts calculated using StringTie (2.1.1)^56^ and the hg38RefSeq gtf attained from UCSC genome table browser August 2020 using default inclusion for All Tracks. Since exon information was not used in generating counts for differential expression, pre-mRNA was not differentiated from spliced mRNA.

#### Differential expression analysis

StringTie counts were imputed into EdgeR and DESeq2 using three comparison structures: Braak Stage I versus II versus III versus IV (six comparisons); Braak Stage I/II versus III versus IV (three comparisons); and Braak stage I/II versus III/IV (single comparison). Since not all entities were protein encoded, we use ‘gene’ to refer to both coding and non-coding annotations. A separate miRNA differential expression analysis used a custom reference gtf that included entries from mirBase and RefSeq.^57^ miRNA was compared across groups using two structures: Braak Stage I/II versus III versus IV (three comparisons) and Braak Stage I/II versus III/IV (single comparison).

#### Functional annotation clustering and gene enrichment

Each gene list was converted to Gene IDs inputted into Database for Annotation, Visualization and Integrated Discovery (DAVID, version 6.8, release October 2016)^58,59^ and processed for annotation clustering (conducted January 2021) using multiple RNA and protein databases with a targeted focus on structure, function and gene ontology (Supplementary Table 1). This software generates an EASE score (one-tail Fisher’s exact probability value), *P*-value^60^ and FDR-corrected value^61^ for each gene and database link within a group and an overall enrichment score for each grouping based on EASE scores.^62^ We used enrichment scores above 1.00 based on the volume of output. Resources used to define gene product interactions and cellular compartment localization included Protein ANalysis THrough Evolutionary Relationships classification system (PANTHER)^63^ and SynGO.^64^ Protein names are derived from UniProtKB retrieved March 2021.

#### miRNA and transcription factor binding site databases

A combination of TarBase v7.0, miRBase and TargetScan databases (retrieved August 2020) generated 2,319 miRNA gene features.^57,65,66^ Annotations from RefSeq, miRbase, and TarBase were crossed and used for downstream analysis. Genes regulated by miRNAs were determined using curated chromatin immunoprecipitation (ChIP)-Seq and experimental data for nucleic acid interactions,^65,66^ and miRNA pathway analysis using the union of genes was performed using DIANA-mirPath.^67^ Significant miRNAs as determined by differential expression analysis for protein-coding genes were excluded from downstream miRNA analyses to avoid cherry-picking data. Transcription factor binding sites (TFBSs) were examined using a combination of CpG islands <300 nucleotides (nt), transcription factor binding clusters observed with ChIP-Seq from the Encyclopaedia of DNA Elements (ENCODE; source data version 2018), and locations with histone 3 acetylated at lysine 27 (H3K27Ac), a marker of active regulatory sites.^68–70^

### Statistical analysis and data visualization

Statistical tests were performed in R (version 4.0.4) and in Excel using custom-designed spreadsheets and scripts. Statistical significance was set at *P* < 0.05 and a false discovery rate (FDR) correction was applied where indicated.^60^ For miRNA differential expression analysis, significance was set at FDR *P* < 0.10. Kruskal–Wallis and χ^2^ tests were used for descriptive statistics and analysis of subject information. Gene counts are presented as counts per million (CPM) based on raw reads normalized within the DESeq2 analysis^71^ adjusted for total reads, and as transcripts per million (TPM) based on a reference-guided assembly step in StringTie.^56^ Also reported is the percentage of subjects in which a gene was expressed (PE) as found through assembly. Gravity network plots were made in Gephi using a two-step gravity loop that applies a separate algorithm at each step with iterations until a limit cycle or steady state is reached. Separate analysis confirmed uniformity with agglomerative hierarchical clustering using traditional distance metrics (hclust, R).

### Data availability

Data are available upon request to the corresponding author and stored in GEO Database. Files document multiple steps in the process to aid further research and cross-validation efforts.

### Results

#### Demographics

Cases were divided into three subgroups: Braak Stages I–II (*n* = 8, 4 M/4F), III (*n* = 8, 3 M/5F) and IV (*n* = 10, 5 M/5F) (Table 1). Although age was significantly different across Braak stages (Kruskal–Wallis *P* < 0.05) no difference was found for MMSE, ApoE genotype, sex, education, post-mortem interval or CERAD criteria (Table 1, Supplementary Fig. 1). Significantly more NIA-Reagan intermediate classifications were found in Braak Stage IV compared with I/II and III (χ^2^  *P* < 0.01, Table 1). A higher proportion of cases displaying a cortical expansion of NFTs (Fig. 1B) was found compared with severity of diffuse and neuritic plaques (Fig. 1C, D) across Braak stages. 6E10 load ranged from absent to moderate in Braak Stages I/II (average score 2.6) and III (2.8) and moderate-to-severe in Stage IV (4.6; Kruskal–Wallis *P* < 0.01, Table 1). Average AT8 load ranged from absent to minimal in Stage I/II (average score 0.6) and III (0.7) and minimal to mild in Stage IV (2.2; Kruskal–Wallis *P* < 0.05, Table 1).

**Table 1:** Subject characteristics

	Braak stage^a^ I–II	Braak stage III	Braak stage IV	χ^2^/Kruskal–Wallis (K)
*n* (male, female)	*n* = 8 (4, 4)	*n* = 8 (3, 5)	*n* = 10 (5, 5)	*P* = 0.84 (χ)
Age at death in years (median)	76–92 (79.9)	82–96 (89.1)	83–93 (86.4)	*P* < 0.05 (K)
Education in years (median)	12–21 (15.0)	14–21 (18.5)	14–27 (19.0)	*P* = 0.31 (K)
MMSE score (median)^b^	25–30 (29.0)	26–30 (28.5)	26–30 (28.5)	*P* = 0.89 (K)
GCS^c^ (median)	(-0.32)-(0.42) (0.113)	(-0.14)-(0.43) (0.264)	(-0.55)-(1.55) (0.141)	*P* = 0.70 (K)
ApoE status	ε2/ε3 *n* = 1	ε2/ε3 *n* = 0	ε2/ε3 *n* = 3	*P* = 0.28 (χ)
	ε3/ε3 *n* = 4	ε3/ε3 *n* = 7	ε3/ε3 *n* = 5	
	ε3/ε4 *n* = 3	ε3/ε4 *n* = 1	ε3/ε4 *n* = 2	
CERAD^d^	definite *n* = 1	definite *n* = 0	definite *n* = 2	*P* = 0.16 (χ)
	probable *n* = 1	probable *n* = 2	probable *n* = 6	
	possible *n* = 2	possible *n* = 1	possible *n* = 0	
	No Alzheimer disease *n* = 4	No Alzheimer disease *n* = 5	No Alzheimer disease *n* = 2	
NIA-Reagan^e^	Intermediate *n* = 1	Intermediate *n* = 2	Intermediate *n* = 8	*P* < 0.01 (χ)
	low *n* = 7	low *n* = 6	low *n* = 2	
PCC 6E10 load ^f^	2.6 (*n* = 8)	2.8 (*n* = 6)	4.6 (*n* = 10)	*P* < 0.01 (K)
PCC AT8 load ^f^	0.6 (*n* = 8)	0.7 (*n* = 6)	2.2 (*n* = 10)	*P* < 0.05 (K)

^a^ Braak staging was deteremined using Bielchowsky silver stain and AT8 immunostaining to identify neurofibrillary tangle (NFT) severity and distribution across the brain. Braak Stages I and II display mild-to-moderate NFTs primarily in the entorhinal cortex; Stages III and IV display a larger involvement into limbic regions including the hippocampus; and stages V and VI revealed moderate-to-severe NFTs across brain regions.

^b^ Mini-mental state examination (MMSE) is a cognitive status examination used to establish a baseline of cognitive function. (no dementia = score 26–30).

^c^ Global cognitive score (GCS) is derived from 19 cognitive test score including episodic memory, semantic memory, working memory, perceptual orientation and perceptual speed performance.

^d^ CERAD (Consortium to Establish a Registry for Alzheimer Disease) based upon post-mortem neuritic plaque pathologic criteria.

^e^ NIA-Reagan [National Institute on Aging (NIA) and Ronald and Nancy Reagan Institute of the Alzheimer's Association (Reagan) consensus diagnosis of Alzheimer's disease].

^f^ PCC (posterior cingulate cortex) average NFT and plaque load scored from 0–absent to 5–severe. Data were not available for two Stage III cases owing to tissue availability.

#### Differential expression data

**Figure 2:**
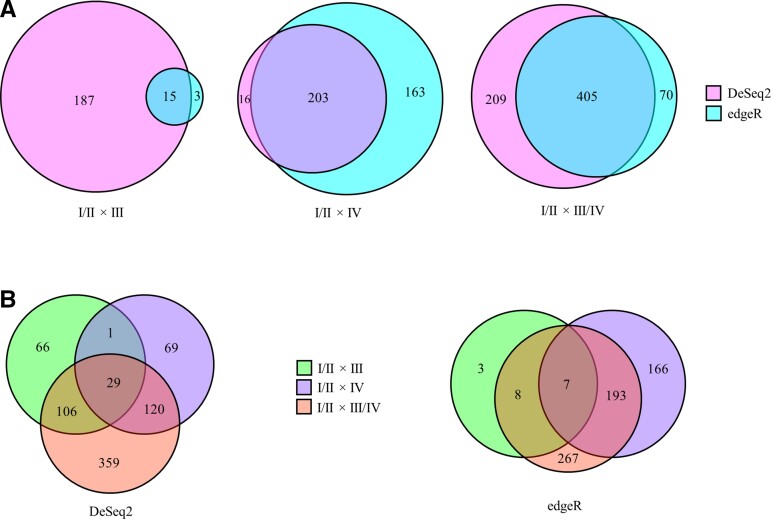
**Venn diagrams showing differences in PCC gene expression across Braak stages.** (**A**) Venn diagrams show a large overlap between DESeq2 and edgeR output. Note that as Braak stage advances overlap is greater, with 7% similarity comparing Stages I/II (*n* = 8) and III (*n* = 8), 53% with Stages I/II and IV (*n* = 10), and 59% with Stage I/II with III/IV. (**B**) Overlap between comparison groups is shown for DESeq2 and edgeR separately. Both bioinformatic tools reveal a large number of genes in comparisons Stages I/II × IV (purple) and I/II × III/IV (red). Only DESeq2 shows a comparable number in comparison Stages I/II × III (green), whereas edgeR analysis found virtually no difference between Braak Stages I/II × III. Neither analysis found any difference with comparison III × IV. Numbers represent DE genes that met the significance FDR cut-off of *P* < 0.05.

Differential expression analysis with edgeR software revealed 644 differentially expressed (DE) genes that were significantly altered in either Braak Stage III or IV or when Stages III and IV were combined (Stage III/IV) and compared with Stage I/II. This contrasts with 750 DE genes found using DESeq2. Combining lists, a total of 917 genes were significantly altered in more advanced Braak stages, meaning 477 genes were shared between the two analyses (Fig. 2). None of the shared genes differed in direction of change. DESeq2 was selected for downstream analysis based on normalized counts, FDR correction and total output.

**Figure 3:**
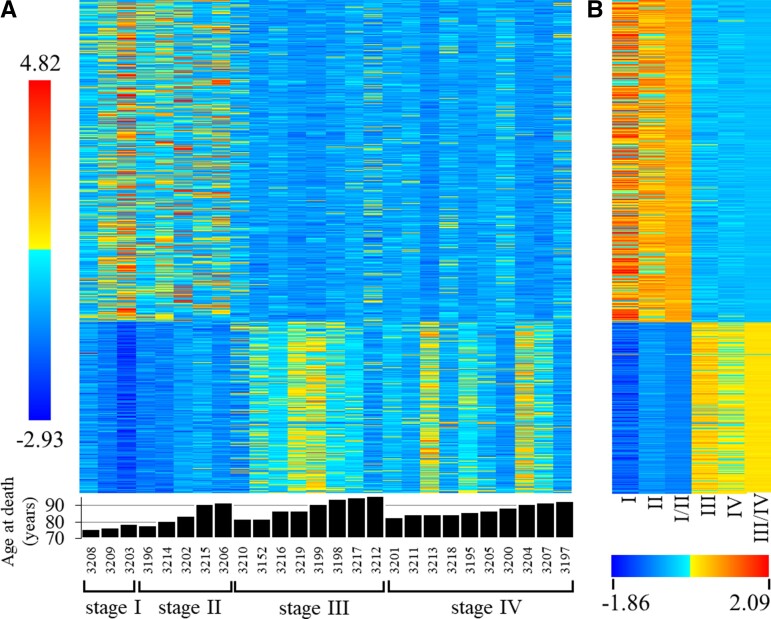
**Heatmaps showing DE genes in the PCC between Braak Stages I/II and III or IV in elderly adults with NCI.** A total of 28,043 features were assessed using DESeq2 to discover DE genes with a FDR cut-off of *P* < 0.05. (**A**) A heatmap shows the results, 489 downregulated genes (top 65% of heatmap) and 261 upregulated genes (bottom 35% of heatmap) in Braak Stages III and IV compared with Stages I and II. Each column of the heatmap represents a person from whom PCC tissue was extracted post-mortem; and each row represents a single DE gene presented through colour-coded *z*-scores calculated using DeSeq2 normalized counts. The colour-scale is shown to the left of the heatmap and represents a range of *z*-scores from 4.82 (red) to -2.93 (blue). At the base of the heatmap, each bar represents the age at death in years of the individuals (represented by blind-coded 4 digit numbers). For example, the first column of the heatmap displays all expression levels for subject 3208, with the subject’s age of 76 years shown below the heatmap on a bar chart. (**B**) A heatmap displays group averages of z-scores used in generating panel A. The colour scale is shown below the heatmap and represents a range of *z*-scores from -1.86 (blue) to 2.09 (red).

Comparison of DE genes that met inclusion criteria for expression level showed that 750 were statistically significant including 489 (65%) downregulated in advanced Braak stages (Fig. 3). In Stage III, 140, Stage IV, 195, and Stage III/IV, 383 genes were expressed at decreased levels compared with Stage I/II. Of these, 26 genes were shared across all three comparisons (Fig. 2B). Finally, 180 genes were decreased only when Stages III and IV were combined, and no differences were observed between Braak Stage III and IV, supporting the variance across these stages (Fig. 3).

Running DESeq2 and edgeR using *APOE* allele status, CERAD, or NIA-Reagan scores did not show a profile similar to that seen with Braak stages. *APOE* allele comparison displayed the following DE genes: DESeq2 revealed *APOE* 2/3 (*n* = 4) had two genes significantly upregulated compared with *APOE* 3/3 (*n* = 16), glycosidase, chitinase 3 like 1 (*CHI3L1*; CPM = 24, TPM = 564, PE = 96%; FDR *P* < 0.05) and immunological response protein, defensin alpha 1 (*DEFA1*; CPM < 2, TPM = 19, PE = 50%; FDR *p* < 0.05) but no DESeq2 genes were significant using edgeR (FDR *p* > 0.72). Since there were only three individuals with a CERAD 1 or CERAD 3 score, we compared CERAD 1/2 (*n* = 12) against CERAD 3/4 (*n* = 14). No DE genes were observed between CERAD groups (DESeq2 FDR *p* > 0.99, EdgeR FDR *p* > 0.76) or NIA-Reagan criteria 3 (*n* = 15) compared with criteria 2 (*n* = 11) (DESeq2 FDR *p* > 0.99, EdgeR FDR *p* > 0.89). A total of 20 DE genes were found between males (XY) and females (XX), 18 were XY homologous genes and 2 have homologues or pseudogenes on sex chromosomes (Supplementary Table 2). There were no differences in *APOE* alleles (χ^2^  *P* = 0.28; Table 1) or expression levels (CPM = 161, TPM = 1102, PE = 100%) across Braak stages. Similarly, no Braak-stage-dependent changes were observed for expression of Alzheimer disease genes *APP* (CPM = 557, TPM = 7616, PE = 100%) or microtubule associated protein tau (*MAPT*; CPM = 732, TPM = 12, PE = 100%). In contrast, the gene for translocase of outer mitochondrial membrane 40 (*TOMM40*; CPM = 23, TPM = 38, PE = 100%) was expressed at higher levels in Braak Stage III compared with Stage I/II (FDR *P* < *0.05*). A complete list of genes of interest is provided in Supplementary Table 3.

### Functional annotation clustering and pathway analysis

#### Downregulation of structure-related transcripts

**Figure 4:**
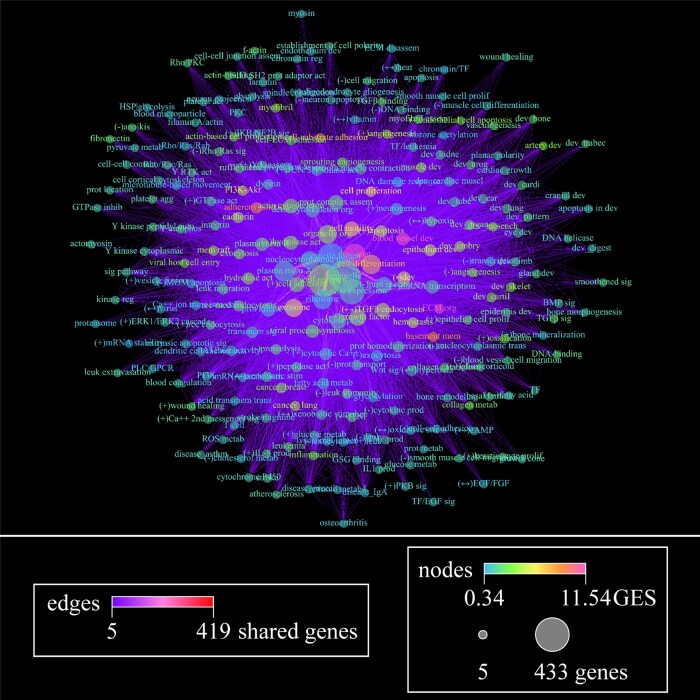
**Weight-directed network plots using functional annotation clustering of differentially downregulated gene expression within the PCC of elderly adults with NCI.** Edges represent genes shared between two functional nodes, with colour demonstrating number of genes shared. Nodes represent functional categories found by annotation clustering using 15 databases. The strength of the relationship between genes in a given node is represented by coloured gene enrichment score (GES). The number of genes contained in each category is represented by the size of the node. Nodes with <5 genes were removed from the network prior to dispersion. Four hundred and eighty-nine genes were downregulated in Braak Stages III or IV compared with Stage I/II, which is represented by 230 nodes and 8,756 edges. A detailed key for node labels can be found in the Supplementary material, and the databases used for ontological enrichment analysis are reported in Supplementary Table 1. (+), upregulation of/within; (-), downregulation of/within; (↔) regulation of/within, direction unspecified.

**Figure 5:**
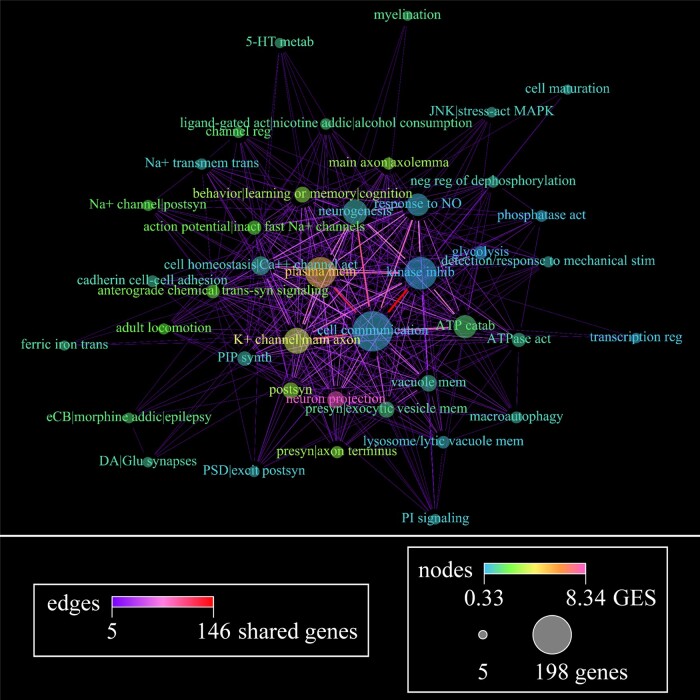
**Weight-directed network plots using functional annotation clustering of differentially upregulated gene expression within the PCC of elderly adults with NCI.** Edges represent genes shared between two functional nodes, with colour demonstrating number of genes shared. Nodes represent functional categories found by annotation clustering using 15 databases. The strength of the relationship between genes in a given node is represented by coloured gene enrichment score (GES). The number of genes contained in each category is represented by the size of the node. Nodes with <5 genes were removed from the network prior to dispersion. Two hundred and sixty one genes were upregulated in Braak Stages III or IV compared with Stage I/II, which is represented by 41 nodes and 374 edges. A detailed key for node labels can be found in the Supplementary material, and the databases used for ontological enrichment analysis are reported in Supplementary Table 1. (+), upregulation of/within; (-), downregulation of/within; (↔) regulation of/within, direction unspecified.

The majority of DE genes in Braak Stages III and IV were downregulated (Fig. 3). Functional annotation clustering based on protein structure, pathway interactions, shared function and gene ontology revealed a predominance of extracellular matrix (ECM) associated genes. Gene subcategories included coding products involved in basement membrane structure and ECM–cell interactions (e.g. adhesion, signalling and cell–cell junctions). Downregulation was seen for genes encoding classes: membrane proteins, vasculature, and protein metabolism. The latter category involved transcripts for peptidases, collagen digestion/absorption and protease inhibitors (Figs. 4 and 5).

#### Synapse gene upregulation

An elevated metabolic profile was found in Braak Stage III and IV compared with Stage I/II. Pre- and postsynaptic channel proteins involved in signal propagation, neurotransmitter release and signal summation were upregulated in advanced Braak stages. The 261 upregulated genes, of which 85 were associated with presynaptic signalling and chemical neurotransmission, were assigned to four functional categories: voltage-gated potassium channel and ion transport at the presynaptic compartment including a link with epilepsy (42 genes, enrichment score 3.13), axon terminus and terminal bouton (18 genes, enrichment score 2.94), anterograde transsynaptic signalling and chemical synaptic transmission; (27 genes, enrichment score 2.52), and pre-synapse and exocytic/synaptic-vesicle membrane (44 genes, enrichment score 1.47). Genes shared across all four of those clusters included glutamate ionotropic receptor NMDA type subunit 2A (*GRIN2A*; CPM = 128, TPM = 1238, PE = 96%) and potassium voltage-gated channel subfamily C member 2 (*KCNC2*; CPM = 55, TPM = 2792, PE = 96%), which encodes the Kv3.2 potassium channel (Table 2).

**Table 2:** Differential expression of miRNA in the PCC in non-cognitively impaired elders

miRNA	Expression level TPM (PE)^a^	Br IV^b^	Br III/IV	Age at death^c^	Working memory	Perceptual speed	Perceptual orientation
hsa-mir-12118	26 (19%)	↓ 17% †	ns	ns	ns	ns	ns
hsa-mir-12121	8 (88%)	ns	↑ 21% †	0.35	ns	ns	0.33
hsa-mir-1302/ hsa-mir-8061	9 (65%)	↓1% †	↓ 1% †	ns	-0.39*	ns	ns
hsa-mir-134	4 (88%)	ns	↑ 16% †	0.56**	ns	ns	ns
hsa-mir-3137	32 (58%)	↑20% ‡	↑ 18% †	0.45*	ns	ns	0.32
hsa-mir-4521	26 (62%)	ns	↓ 21% †	-0.40*	ns	0.42*	ns
hsa-mir-4528	4 (42%)	ns	↑ 14% †	0.42*	ns	ns	0.39
hsa-miR-4639-3p/	< 2 (35%)	↑9% ‡	ns	ns	ns	ns	ns
hsa-mir-548a-3p/							
MIR548A1HG							
hsa-mir-4705	916 (92%)	ns	↑ 18% †	0.42*	ns	ns	0.48*
hsa-mir-548aj-5p/	303 (100%)	ns	↓ 6% †	-0.31	ns	0.40 *	ns
MID1IP1							
hsa-mir-5692b	17 (73%)	↑ 25% ‡	↑ 22% †	0.45*	ns	ns	0.46*
hsa-mir-617	26 (62%)	↑ 21% †	↑ 18% †	ns	ns	0.31	0.33

^a^ TPM transcripts per million calculated after reference-guided assembly in StringTie (2.2.1); PE, percent of subjects expressed within.

^b^ Percentage change (↓, downregulation; ↑, upregulation) compared with Braak Stages I/II. No significant differences were found between Braak Stage IV and III or Braak Stage III and I/II.

^c^ No significant correlations were found for the subject information: years of education, mini-mental state examination, global cognitive score, episodic memory and semantic memory.

^†^ FDR *P* < 0.10; ‡ FDR *P* < 0.05; * *P* < 0.05; ** *P* < 0.01; ns, not significant.

Genes associated with the post-synapse were also affected, with 50 genes upregulated in Braak Stages III and IV compared with I/II divided into two functional categories: postsynaptic membrane (44 genes, enrichment score 3.23) and voltage-gated sodium channel activity/postsynaptic membrane depolarization (12 genes, enrichment score 2.09). Five genes were shared across groups: calcium voltage-gated channel auxiliary subunit beta 4 (*CACNB4*; CPM = 32, TPM = 2898, PE = 100%), *GRIN2A*, sodium voltage-gated channel alpha subunit 1 (*SCN1A*; CPM = 123, TPM = 2170, PE = 100%), sodium voltage-gated channel beta subunit 4 (*SCN4B*; CPM = 9, TPM = 795, PE = 100%) and solute carrier family 17 member 6 (*SLC17A6*; CPM = 5, TPM = 58, PE = 100%), which encodes the presynaptic vesicular transporter for glutamate VGLUT2. Hence, a profile of increased excitatory neurotransmission and membrane depolarization emerged at more advanced Braak stages.

#### Energy metabolism expression

Braak Stages III and IV had elevated expression of mRNAs enriched for three ATP-related functional clusters including genes encoding presynaptic synaptojanin 1 (*SYNJ1*; CPM = 223, TPM = 73, PE = 100%). Six genes encoded regulatory proteins including postsynaptic kinase modulator, protein kinase cAMP-dependent type II regulatory subunit beta (*PRKAR2B*; CPM = 49, TPM = 1233, PE = 92%). Genes encoding proteins involved in ion channel or transporter function included pre- and postsynaptic ATPase plasma membrane Ca2 + transporting 2 (*ATP2B2*; CPM = 252, TPM = 10,285, PE = 96%), hyperpolarization activated cyclic nucleotide gated potassium channel 1 (*HCN1*; CPM = 23, TPM = 893, PE = 96%) and presynaptic potassium voltage-gated channel subfamily H member 1 (*KCNH1*; CPM = 39, TPM = 480, PE = 100%). A gene encoding a presynaptic protein involved in membrane trafficking, N-ethylmaleimide sensitive factor, vesicle fusing ATPase (*NSF*; CPM = 232, TPM = 105, PE = 100%) was associated with two of the three categories, further supporting elevated synaptic activity in NCI subjects with higher Braak stages.

#### Transcription regulatory mechanisms

Downregulation of transcription-associated genes was seen in Braak Stages III, IV and III/IV combined compared with Stage I/II including four functional/structural categories and seven annotation clusters: domain LIM and zinc-binding (7 genes, enrichment score 2.52); domain WW (6 genes, enrichment score 2.52); RNA polymerase II TF activity and sequence-specific DNA binding transcription factor forkhead box (FOX) (15 genes, enrichment score 1.90); and positive regulation of transcription from RNA polymerase II promoter (97 genes, enrichment score 1.64; 307 genes, enrichment score 1.54; 134 genes, enrichment score 1.22); and regulation of transcription from RNA polymerase II promoter and negative regulation of protein metabolic process (seven genes, enrichment score 1.40). A total of 17 genes were seen in five of the seven clusters, including two involved in chromatin modelling, 13 involved in gene-specific transcription regulation and 2 coded for cell structure products.

**Figure 6:**
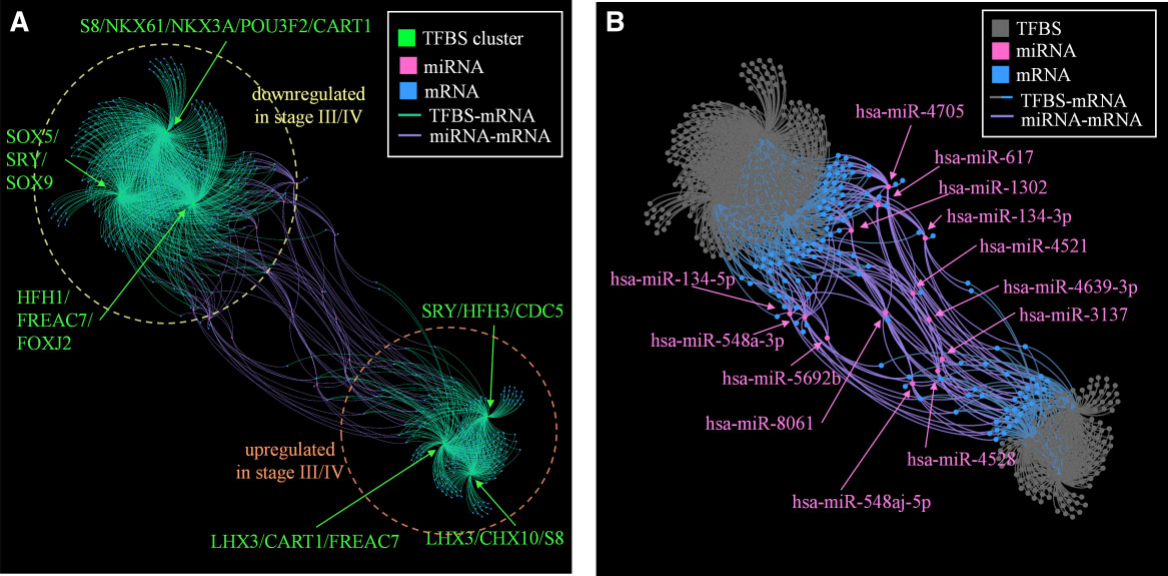
**Association networks showing relationship of regulatory mechanisms and DE genes in Braak Stages I/II compared with Stages III/IV within the PCC of elderly adults with NCI.** Functional annotation clustering of 750 DE genes was performed using a TFBS annotation file that combines information on chromatin structure and ChIP (see Methods) to derive a list of genes associated with a given TFBS. Clustering first matches Braak-stage DE genes to respective associated TFBSs and groupings of TFBS are selected using a calculated enrichment score (based on number of DE genes) to determine significance. Following discovery of significant TFBS clusters, we used a network-based map to illustrate associations. (**A**) Three TFBS clusters were associated each with DE genes with expression significantly upregulated and downregulated in Braak Stage III/IV compared with I/II. Of note, the direction of expression change refers exclusively to DE genes, and not TFBS factors. This plot is spatially agnostic and no information can be derived from axes; the layout is the consequence of a force-directed algorithm and conveys information only in distance (farther = looser association), not in position relative to any constant (like an axis or grid). Each green dot (node) represents a TFBS cluster (e.g., LHX3/CART/FREAC7); blue, a specific expressed gene (mRNA) with higher (bottom right) or lower (top left) levels in Braak Stages III/IV compared with Braak Stages I/II; and, pink, a specific microRNA. Every line (edge) represents an association as determined from the TFBS database outlined previously, coloured according to component nodes with no information delivered via edge thickness. Node size is based on number of associations but should be considered minimally informative at this resolution. All associated mRNA can be found in Supplementary Fig. 2A. (**B**) DE microRNA (miRNA), as detected in a separate differential expression analysis, were analyzed for associated genes through a literature and multiple database search (see Methods). This compiled list of associated genes was then crossed with the DE genes. Interestingly, many mRNA nodes have multiple associations with regulatory TFBS and miRNA. The direction of change for miRNA had no consistent association with direction of change in mRNA (Supplementary Fig. 2B, C) and many miRNA were associated with up- and downregulated mRNA seemingly indiscriminately. Names of genes associated with miRNA can be found in the Supplementary material.

A combination of DNA structure, sequence identity and ChIP-Seq data from curated databases found clustering of multiple TFBSs associated with gene profiles downregulated in Braak Stages III and IV compared with I/II (Fig. 6). An independent differential expression analysis specific to a list of > 2,000 miRNAs revealed three miRNAs upregulated in Braak Stage IV compared with Stage I/II (*P* < 0.10, Table 2) and three miRNAs downregulated in Braak Stage IV compared with Stage I/II (*P* < 0.10, Table 2). Combining Braak Stages III/IV revealed seven upregulated and three downregulated miRNAs compared with Stage I/II (Table 2, Supplementary Fig. 2). Crossing miRNA lists with a miRNA-specific pathway databases revealed significant gene intersection for phosphatidylinositol signalling [19 miRNA-database (miR-db) hits, FDR *P* < 0.001], endocytosis (19 miR-db hits, FDR *P* < 0.005), axon guidance (15 miR-db hits, FDR *P* < 0.00001), glutamatergic synapse (14 miR-db hits, FDR *P* < 0.005), long-term potentiation (13 miR-db hits, FDR *P* < 0.005), nicotine addiction (12 miR-db hits, FDR *P* < 0.001) and extracellular structure pathways (adherens junction, 18 miR-db hits, FDR *P* < 0.00001; proteoglycans, 17 miR-db hits, FDR *P* < 0.00001) (Supplementary Table 4).

#### Dimensionality reduction highlighted gene upregulation

PCA of the 750 DE genes explored covariance within individuals. Dimension 1 accounted for 46.2% of the variance and Dimension 2 10.4%. After running the regression calculation using DESeq2 normalized gene expression values, we examined subject factors not used in deriving the PCA results. This process collapses subjects within categorical groupings (e.g., Braak stage) to derive a theoretical variable location and confidence interval, presented as coordinates and ellipsis on a Dimension 1 × Dimension 2 biplots. Although Braak Stage I/II segregated from Stages III and IV, there was no difference between Braak Stages III and IV (Supplementary Fig. 3). Overlay of male/female (categorical), APOE ε status (categorical) and age at death (vector) did not show differences across categories or influence of age on a biplot, or very near the origin, indicating that age lies in a different dimension (Supplementary Fig. 3).

Although 65% of the DE genes were downregulated in Stages III and IV, PCA using the same gene list highlighted upregulated genes as the largest contributor to variance across subject gene expression profiles. Taking the top 10% of contributors to Dimension 1 (*75* genes accounting for 22.7% of Dimension 1, and 10.5% of total variance), 69 genes (92%) were upregulated and six genes were downregulated in Braak Stages III and IV compared with Stage I/II, a stark difference from the 35% percent of total DE genes that were upregulated in the more advanced Braak stages. Functional clustering highlighted neuronal cation channel activity (48 genes, enrichment score 2.88), synaptic signalling (10 genes, enrichment score 2.06), and postsynaptic membrane (15 genes, enrichment score 2.00) as pathways and physiological mechanisms enriched in Dimension 1 upregulated genes, whereas phosphoprotein binding (six genes, enrichment score 1.08) and transcription regulation (four genes, enrichment score 1.02) were enriched in the Dimension 1 downregulated genes.

PCA revealed neither a contribution by cognitive domain and performance scores nor highlight a difference between Braak stage I/II and Braak stage III or IV. Dimension 1 on the cognitive PCA contributed 27.7%, and Dimension 2 13.4%, which is closer to a random distribution (based on a run of 10 PCA with values from cognitive data replaced with random numbers, average Dimension 1 = 12.5%, Dimension 2 = 11.2%, regression slope 0.64) than to the PCA with genes. There was a difference between male and female theoretical variable overlays on the cognitive PCA (Supplementary Fig. 3C, D). As with the gene expression PCA, age and APOE ε status did not show an influence on Dimension 1 × Dimension 2 biplots.

#### Synapse-related functional pathways associate with cognitive performance

Significant DE genes correlated with cognitive test scores (Table 3) following FDR correction for multiple comparisons. A cut-off of rho ≥ |*0.55*| with an uncorrected *P*-value *<0.005* was used to determine associations. Gene expression was not associated with composite cognitive scores for episodic, working, or semantic memory (Table 3). Less than 1% of DE genes (< 8 genes) correlated with each episodic memory test: delayed logical memory II, word list and word list recall; working memory test: alpha span; and semantic memory tests: category fluency and reading test. Ten genes positively and one negatively associated with performance on the Boston naming test of semantic memory. Of note, the 10-item reading test was associated with 5 genes, including neurotrophic receptor tyrosine kinase 1 (*NTRK1*; CPM <2, TPM <2, PE = 100%), which encodes TrkA, the cognate receptor for nerve growth factor (NGF).^72–74^

**Table 3:** Correlations between cognitive performance scores and gene expression in non-cognitively impaired elders

Cognition domain/test^a^	KW p^b^	corr dir^c^	Genes involved gene symbol (Spearman rho^c^)
MMSE	0.89	na^d^	
Global cognitive functioning	0.70	na	
Episodic memory	0.95	na	
Logical memory II (delayed)	0.67	+	TMPRSS13 (0.55)
East Boston delayed recall	0.99	na	
East Boston immediate recall	0.89	na	
Logical memory I (immediate)	0.39	na	
Word list	0.56	+	ADPRH (0.55)
Word list recall	0.69	-	C1orf158 (-0.55)
Word list recognition	0.27	na	
Working memory	0.33	na	
Alpha span	0.32	+	BAMBI (0.60), DRC7 (0.58), LOC100507412 (0.62), REG4 (0.72)*, SLAMF1 (0.56)
		-	HAR1A (-0.58), TECPR1 (-0.57), TMEM191A (-0.56)
Digit ordering	0.05	na	
Digits backward	0.08	na	
Digits forward	0.57	na	
Semantic memory	0.78	na	
Boston naming (15 items)	0.16	+	AHNAK (0.56), ERBB2 (0.57), F2R (0.55), MORC4 (0.58), MYLK (0.60), OCLN (0.55), TGFB1I1 (0.57), TNS1 (0.57), ZBTB20-AS1 (0.56), EYA1 (0.59)
		-	DPY19L2P4 (-0.59)
Category fluency (fruits)	0.81	-	CCDC170 (-0.55)
Extended range Vocabulary	0.47	na	
Reading test (10 items)	0.25	+	NTRK1 (0.58), PAX1 (0.56), SLAMF1 (0.57), TMPRSS13 (0.59)
Perceptual orientation (visuospatial ability)	0.19	+	AACS (0.58), CCDC85A (0.58), CLSTN1 (0.56), CLVS2 (0.63), CNTNAP1 (0.60), EPDR1 (0.64), FAM135B (0.63), FRRS1L (0.56), HCN1 (0.56), INPP5F (0.56), KCNA2 (0.57), KCNC2 (0.67), KLHL18 (0.65), LANCL3 (0.57), LINC02035 (0.63), LOC100287846 (0.55), LPCAT4 (0.56), LSM11 (0.62), MADD (0.60), MAPK9 (0.59), MCF2 (0.60), NAA30 (0.58), NDRG4 (0.59), NDUFAF5 (0.59), OGDHL (0.56), PDK3 (0.65), PEG13 (0.58), PIP4K2C (0.58), PNMA1 (0.55), PPP1R14C (0.57), PRICKLE1 (0.57), PWAR5 (0.55), PWARSN (0.59), RFPL1S (0.56), RNF175 (0.56), RTN1 (0.58), SACS (0.63), SCN4B (0.57), SCN8A (0.58), SLC3A1 (0.55), SLC9B2 (0.61), SNHG14 (0.63), SS18L1 (0.57), SYNJ1 (0.58), TAF4B (0.59), TMEM35A (0.59), TPX2 (0.68), TRPC5 (0.64), UBE2O (0.55), XK (0.58), ZNF204P (0.60), ZNF483 (0.60)
		-	ARHGEF5 (-0.55), ATAD2B (-0.62), BMP7 (-0.61), C14orf93 (-0.55), DENND2C (-0.69)*, DIPK2B (-0.75)*, EGFLAM (-0.56), EPHX1 (-0.55), FOXD2-AS1 (-0.62), HEG1 (-0.66), HEY2 (-0.58), LOC100507053 (-0.57), MAML2 (-0.58), NKD1 (-0.60), PAQR5 (-0.58), POFUT1 (-0.59), SOX13 (-0.57), SPN (-0.63), TGFBR2 (-0.59), TRIM34 (-0.56), UACA (-0.62), USP39 (-0.55), WWTR1 (-0.64)
Line orientation	0.38	+	FAM217B (0.57), LANCL3 (0.57), PPP1R14C (0.61), PWAR5 (0.60), PWARSN (0.58), XK (0.57), ZNF483 (0.59)
		-	ACVRL1 (-0.56), HEG1 (-0.59), MAML2 (-0.64), MYOF (-0.58), PLP2 (-0.57), PRELP (-0.59), SOX13 (-0.59), SPN (-0.63), TINAGL1 (-0.65), TLN1 (-0.56), WWTR1 (-0.64), ZFP36L1 (-0.65)
Progressive matrices (16 items)	0.40	-	CD28 (-0.61), COL6A3 (-0.57), GOLGA8G (-0.58), IL36B (-0.58), LINC02476 (-0.56), LOC100507053 (-0.63), NR1H4 (-0.58)
Perceptual speed	0.52	+	DBET (0.59)
		-	HAR1A (-0.56)
Number comparison	0.89	na	
Symbol digits modality-oral	0.28	+	CCDC33 (0.60), DBET (0.68)

^a^ Median time from last testing date to death is 7.6 months.

^b^ Kruskal–Wallis test for significance across Braak Stages I/II, III and IV.

^c^ Direction of correlations.

^d^ Only correlations ≥ |0.55| are presented, all correlations were at least *P* < 0.005; however, the BH burden was 0.000067; asterisk (*) and bold-face show correlations significant with the FDR correction.

^e^ na, no associations that met criteria.

Two component subtests related to perceptual orientation were associated positively with 53 and negatively with 36 genes. Of the latter, 2 genes met FDR criterion. DENN domain containing 2C (*DENND2C*; CPM < 2, TPM = 14, PE = 100%), a positive regulator of GTPase activity involved in vesicle-mediated trafficking, was significantly decreased by 36% in Braak Stage III/IV compared with I/II and divergent protein kinase domain 2B (*DIPK2B*; CPM = 2, TPM = 117, PE = 100%), an X chromosome gene with links to autism,^75^ was decreased by 80% in Braak Stage III/IV compared with I/II. Functional annotation clustering based on gene structure, function and gene ontological category using genes positively correlated with the composite perceptual domain score showed enrichment in transcript classes encoding proteins associated with axon activity and postsynaptic membrane potential (Supplementary Tables 5 and 6).

## Discussion

We found 489 downregulated and 261 upregulated genes in PCC obtained from elderly subjects that died with a pre-mortem clinical diagnosis of NCI and post-mortem pathological evaluation of Braak Stage I, II, III and IV. Despite predominantly downregulation across Braak stages, upregulation of individual expression profiles was most prevalent in Stage III and IV compared with I/II. Dimension reduction analysis found that upregulated genes primarily contributed to Dimension 1, which accounted for nearly half of the covariance across individuals. Of the top 10% Dimension 1 genes, enrichment was primarily related to excitatory synaptic transmission, which correlated strongly with cognitive performance. Dimension 2, the next highest orthogonal contributor to individual covariance, revealed a decrease of neuromodulatory genes in later Braak stages with differences between Braak Stages III and IV. These novel findings emphasize the profound changes in synaptic and neuromodulatory genes that may underlie a mechanism of resiliency and cognitive reserve in the face of mounting Alzheimer disease pathology with NCI. Commensurate with our post-mortem human brain findings, animal models of aging have been integral in the development of a compendium of possible candidates for cognitive reserve genes (CRGs).^76–78^ Further, independent studies that support the current results found gene expression alterations between Braak stage I/II compared to III that were related to synaptic plasticity, mitochondrial function, GPCR signalling, electron transport and calcium ion binding, among others in the prefrontal cortex (PFC) DMN hub.^79,80^

Upregulation of genes encoding synaptic transmission and cellular energy metabolism observed in the more advanced Braak cases is analogous to increased frontal lobe neuroactivity reported in older adults without cognitive impairment measured by PET imaging.^81,82^ These findings suggest that these alterations are involved in the compensatory preservation of cognition despite the increase in neuropathology. Over time, these initial cognitive resilience mechanisms to maintain function may ultimately fail to preserve cognition with advancing age or are overwhelmed by the onslaught of disease pathology.^83^ For example, the present findings suggest resiliency at the metabolic level may fail in those with cognitive decline similar to that seen in model organisms.^83^ In addition to aging and pathology, resilience likely is influenced by sex, life experiences, education, connectional plasticity, and epigenetics.^83–86^ Whether upregulation in cellular activity genes underlying metabolic dysregulation and altered connectivity patterns found in the PCC across Braak stages is similar or different between hubs of the DMN remains to be determined.^32,33,87,88^ Therefore, uncovering the mechanism(s) for increased cortical synaptic activity will have clinical and quality-of-life implications for the elderly and enhance putative therapeutic implications using previously reported novel CRGs.^78,89^

There are no PCC transcriptomic datasets in elders across the Alzheimer disease spectrum that offer a tool for comparison, highlighting the importance and novelty of the present findings. Analogous PFC gene expression in pre-middle-aged (≤ 40 years) compared to aged non-demented adults (≥ 70 years) found decreases in genes associated with inhibitory neurotransmission and neuropeptide systems.^90^ Interestingly, while GABA marker gamma-aminobutyric acid type A receptor subunit gamma2 (*GABRG2*) and glutamate marker G protein-coupled receptor 158 (*GPR158*) expression were increased in the PCC of Braak Stage III/IV compared with I/II, a significant downregulation occurred in the PFC of aged compared with pre-middle-aged adults.^90^ Although this may represent regional DMN profile differences, a pathology × age interaction could relate to cohort composition or size. *GABRG2* and *GPR158* expression levels in the brain^91,92^ is linked to aging^93,94^ and adult neuropsychiatric conditions,^95–97^ and *GPR158* expression is associated with Alzheimer disease pathology as well as frontotemporal dementia.^98,99^ Moreover, *GPR158* downregulation is related to hippocampal-mediated cognitive deficits.^93,100,101^ Interestingly, glutamatergic presynaptic markers increase in MCI cortex, suggesting a paradoxical inhibitory response to dementia onset.^102^

Of the pre- and postsynaptic protein-encoding genes upregulated in Braak Stages III and IV compared with I/II, *GRIN2A* mRNA is also elevated in the hippocampus in MCI compared with NCI^103^ suggesting a target for intervention.^104^ Microarray studies also reveal *VAMP1* mRNA elevation in the superior frontal gyrus and increased hippocampal *STXBP5L* mRNA in MCI compared to NCI, while both are decreased in entorhinal cortex,^103^ suggesting differential brain regional vulnerability between aging and the onset and progression of Alzheimer disease. A negative association of *VAMP1* expression and Braak stage was observed when analysis included Braak Stage V and VI.^105^ Upregulation of these genes was found in PCC of Braak Stage III/IV compared with I/II, with no advanced stages for comparison. While altered exocytotic vesicle transcripts along with *VAMP1* and *STCBP5L* occur in the hippocampus and PFC in MCI compared with NCI,^103^ similar findings were not seen in our study. Notably, studies using lower organisms report opposing directional changes in transcripts and proteins in response to pathological mutations.^106^ We found similar decreases to those reported in MCI compared with NCI including decrements in neocortical expression for *ITGB1* and *ITGB8*.^103^ Therefore, ITGB1 may play a role in the progression of Alzheimer disease through alterations in oxidative stress.

Increased expression of postsynaptic genes reveals elevated synapse activity and a decrease in neuromodulatory genes in more advanced Braak stages. Genes encoding vesicular transporters for dopamine, DAT (*SLC6A3*), and norepinephrine, NET (*SLC6A2*), involved in the synaptic reuptake of catecholamine neurotransmitters, as well as choline acetyltransferase (ChAT), the synthetic enzyme for acetylcholine were significantly decreased in PCC in Braak Stages III/IV compared with I/II. Although decrements in ChAT activity have been reported in the PCC in Alzheimer disease, ChAT expression remains stable in MCI.^107^ Interestingly, we found upregulated excitatory gene profiles even within functional clusters defined by neuromodulatory circuits. For example, we found an increase in VGLUT1 (*SLC17A6*), a presynaptic transcript that encodes a protein involved in primary excitatory transmitter release, and a decrease in the transcript that encodes a transporter involved in Glu synthesis xCT (*SLC7A11*) in functional clusters associated with the neurotransmitters, dopamine, noradrenaline, and serotonin. These findings suggest a molecular signature of decreased neuromodulatory activity and elevated excitatory neurotransmission. Examining these changes in light of neuropathology, changes in genes encoding DAT, NET, and ChAT occurred in Braak stage IV, whereas excitatory transmitter changes were seen in Stages III or III/IV. This provides a possible timeline for resilience through molecular mechanisms whereby neuromodulation is altered in response to elevated excitatory neurotransmission. Since the PCC receives neuromodulatory innervation from spatially distinct cell populations, this raises the possibility of a diffuse connectome reorganization in NCI elders with increased NFT pathology. These observations may demonstrate neuroplasticity associated with resilience that may play a role in the ability to perform age-related task completion strategies.^81,82^

We found significant differences in miRNAs only in later Braak stages (e.g. IV or III/IV compared with Stage I/II) and no detectable differences at Stage III compared with Stage I/II. These findings support previous studies suggesting miRNA alterations occur later than alterations in genes they regulate in individuals with MCI compared to NCI.^108^ Possible factors contributing to these temporal differences include a secondary regulatory response to disease onset or an inability to regulate homeostasis by post-translational modifications,^109^ an idea supported by our finding Braak Stage III changes in gene pathways involved in transcription regulation. Further, Braak Stages III and IV show a marked upregulation in transcripts encoding kinases, downregulation in phosphatases, and an increase in ubiquitin protein-encoding, pathways similarly implicated in Alzheimer disease.^110,111^ Alterations of protein metabolic factors also occur in MCI compared with aged controls^103^ and are associated with NFTs,^112^ differentiating these changes from normal aging.^113^ The changes in miRNAs associated with Braak Stage IV indicate an expression imbalance in response to pathogenesis and may provide a viable target for identifying resilience or lack thereof across the Alzheimer disease spectrum.^114^

The specific miRNA alterations reported here have not been previously identified across the Alzheimer disease spectrum; however, there is poor consensus and systematization for the evaluation at non-coding regulatory elements, making comparison tenuous.^115,116^ When we crossed the miRNA list with known gene interactions, we found DE genes more highly expressed in Braak Stages III and IV were those involved in synaptic activity, especially with regards to regulatory elements hsa-miR-8061 and hsa-miR-548a-3p, miRNA decreased in Braak Stage IV, and hsa-miR-5692b and hsa-miR-134-5p, miRNA increased in Braak Stage IV compared with Stage I/II. Moreover, comparison of miRNA expression with cognitive function revealed high association with the same visuospatial domains associated with DE genes found in a separate analysis. The precise molecular pathogenic role that miRNAs play during the progression of Alzheimer dementia remains to be defined. As exploration into CRGs continues, these regulatory mechanisms may prove insightful for defining a timeline and therapeutic targets for the treatment of cognitive decline in the elderly and those with dementia.^78^

PCC expression profiling revealed a significant association with NFTs but not amyloid or neuritic plaque pathology. Differential expression analysis using CERAD or NIA-Reagan neuropathological scores as grouping factors was indistinguishable from random grouping. This corresponds with prior investigation of individuals with MCI or Alzheimer disease that demonstrated minimal correlation between parenchymal plaque pathology and cognitive impairment.^17,117^ Similarly, ApoE allele as a grouping factor was indistinguishable from a random grouping factor on differential expression analysis and PCAs with either gene expression or cognitive performance. However, a study of ApoE status and brain glucose metabolism in non-demented adults aged 30–95-years-old found an age-related significant decline with greater uptake in ε4 noncarriers compared with carriers in DMN hubs including the PCC.^118^ Moreover, in participants older than 70 years, there was no interaction between Pittsburgh Compound B amyloid binding status and APOE ε4 genotype with respect to glucose metabolism.^118^ These findings indicate the PCC has a unique vulnerability to reductions in glucose metabolic rate as a function both of age and APOE allele status, perhaps due to its role as a hub of the DMN that deactivates when mental effort is required but is less efficient in deactivation during the progression of Alzheimer disease.^118^ Since ApoE genotype represents a life-long state, persons with a higher level of education or a lifestyle that involves frequent cognitive engagement may be less likely to have detectable differences on cognitive tests that correlate with ApoE allele status. A potential limitation in the present study is that the small number of ε4 carriers may mask PCC genotype changes associated with ApoE ε status.

It is possible that educational level affects the expression of various classes of transcripts including the upregulation of synaptic genes. Level of education has been suggested to play an important role in preventing the onset of dementia through brain reserve.^119^ Interestingly, the higher Braak stage group had an average education level 4 years greater than the lower Braak cases, suggesting the intriguing concept that educational level plays an active role in the upregulation of synaptic transcripts found in the current high Braak cases.^119^ More detailed investigations of the interaction between education, Braak stage, brain resilience, and gene expression are warranted.

Studies indicate *TOMM40* variants are associated with estimating onset of Alzheimer disease and interaction with ApoE status can increase disease onset, which may be geographically dependent.^120^ We found that *TOMM40* expression was significantly increased in Braak stage III compared to I/II with no difference in transcript variants based on reference-guided assembly. Previously, blood analysis revealed a significant association between longer *TOMM40* poly-T lengths and neuroimaging higher medial temporal cortex plaque and NFT burden in non-demented older adults.^121^  *TOMM40* ‘523 polymorphism affects expression levels of APOE, and *TOMM40* mRNAs in the temporal and occipital cortices of late-onset Alzheimer disease and non-demented controls.^122^ The molecular and biochemical mechanism(s) underlying the effect of increased *TOMM40* expression upon Alzheimer disease pathophysiology remains to be investigated. However, structural DNA variations, especially those in intronic or intergenic regions such as *TOMM40* ‘523, may alter gene transcription efficiency, timing of transcription, transcript stability, transcript splicing and/or epigenomic modifications.^123^ While we have studied transcript variants from reference-based assembly, we have not yet investigated polymorphisms. This is in progress for all DE genes and will help to clarify the possible role of *TOMM40* in CRG-related processes. However, it is possible that *TOMM40* is part of a resilience mechanism that is specific to a select group of variants and not the generalized elderly population.

We provide evidence for putative brain cognitive reserve as a mechanism for resiliency based upon differential molecular expression profiling of the PCC genes derived from elders with NCI but with different Braak scores.^114,124^ Although the present definition is similar to that established by the Collaboratory on Research Definition for Reserve and Resilience, it also is reminiscent of ‘potential cognitive reserve genes’, in which genes are selected depending upon whether they display differential expression^78^ based upon Braak stage. In the present report, brain resilience and cognitive reserve suggest that a population older individuals have functional and structural physiological changes, such as increased synapse number or size, or adjusted cognitive strategies which allow the brain to tolerate a greater degree of pathology without suffering decline on cognitive tasks.^114,125^ Along this line, resiliency/reserve may also involve recruitment of other brain areas resulting in increased cortical innervation from regions not severely affected to aid in task performance. Our findings suggest that cognitive reserve and resilience likely involves synaptic and metabolic pathway expression that increases across Braak Stages III and IV as a potential compensatory response to age-related cortical denervation.^126^ In this regard, it has been proposed that reserve can be measured or inferred either through increased brain structural and/or physiological pre-morbid capacity.^127^ Interestingly, a disconnect between the Alzheimer disease proteome and transcriptome in the PFC was reported,^128^ suggesting the importance of investigating proteins in addition to their coding transcripts that likely play a role in brain resilience, especially within hubs of the DMN including the PFC and PCC. Interestingly, a mathematical assessment of the transcriptome from different aging studies found in relevant animal models one in ∼six age-related genes were considered poor behavioural predictors, highlighting expression variability and biological variance^78^ that may be applicable to defining CRGs and exploiting them for therapeutic interventions.

Finally, it is important to consider study limitations. Tissue was obtained from a subpopulation of the RROS cohort with lifestyle elements that differ from a secular community-based cohort,^129,130^ which likely affect the bidirectional relationship between cognitive stimulation and cognitive status.^125,131–133^ Since we examined individuals who aged into their 9th decade without cognitive impairment, natural limitations affect cohort size and applications of computational detection allowing for clustering into expression between successful agers versus those progressing to MCI.^134^ However, a strength of this population is homogeneity and low rate of subject attrition over time. Importantly, regional brain dissections consist of an admixture of different cell types resulting in an expression profile that masks changes in specific cells at the sequencing and computational stage. Notwithstanding these caveats, we uncovered mRNAs in human PCC that were differentially expressed between Braak Stages I/II and III/IV in addition to associated miRNAs and TFBSs. Inputting significantly different gene/network data into a functional annotation clustering model revealed elevated presynaptic, postsynaptic and ATP-related expression in Braak Stages III and IV compared with Stages I/II, suggesting these pathways are integral for cognitive resilience seen in elderly non-demented cases. Braak stage was not associated with cognitive function but upregulation of synaptic genes positively correlated with visuospatial perceptual orientation tasks. These findings suggest increased synaptic expression, in part, underlies cognitive resilience in elders despite Alzheimer disease pathology.

## Supplementary Material

fcac162_Supplementary_Data

## Abbreviations

Aβ =: amyloid-beta peptide
APOE =: apolipoprotein E
APP =: amyloid-beta precursor protein
CERAD =: Consortium to Establish a Registry for Alzheimer Disease
ChIP =: chromatin immunoprecipitation
CPM =: counts per million
CRGs =: cognitive resilience genes
DAVID =: Database for Annotation, Visualization and Integrated Discovery
DE =: differentially expressed
DMN =: default mode network
ECM =: extracellular matrix
ENCODE =: Encyclopaedia of DNA Elements
FC =: frontal cortex
FDR =: false discovery rate
FLASH =: Fast length adjustment of short reads
GCS =: global cognitive score
H3K27Ac =: histone 3 acetylated at lysine 27
IPC =: inferior parietal cortex
MCI =: mild cognitive impairment
miRNA(s) =: microRNA(s)
miR-db =: miRNA-database
MMSE =: Mini-Mental State Examination
MTL =: medial temporal lobe
NCI =: no cognitive impairment
NFT(s) =: neurofibrillary tangle(s)
NGF =: nerve growth factor
NIA-Reagan =: National Institute of Aging/Reagan Institute
nts =: nucleotides
PANTHER =: Protein ANalysis THrough Evolutionary Relationships classification system
PCC =: posterior cingulate cortex
PE =: percent expressed
RNA-Seq =: RNA sequencing
RROS =: Rush Religious Orders Study
STC =: superior temporal cortex
TF(s) =: transcription factor(s)
TFBS(s) =: transcription factor binding site(s)
TOMM40 =: translocase of outer mitochondrial membrane 40

## References

[1] Facts and Figures. Alzheimer's Association . Updated 2021. https://www.alz.org/alzheimers-dementia/facts-figures

[2] Jack CR J, Knopman DS, Jagust WJ, et al Hypothetical model of dynamic biomarkers of the Alzheimer's pathological cascade. Lancet Neurol. 2010;9:119–128. 10.1016/S1474-4422(09)70299-6 20083042 PMC2819840

[3] Jicha GA, Parisi JE, Dickson DW, et al Neuropathologic outcome of mild cognitive impairment following progression to clinical dementia. Arch Neurol. 2006;63:674–681. 10.1001/archneur.63.5.674 16682537

[4] Petersen RC, Wiste HJ, Weigand SD, et al Association of elevated amyloid levels with cognition and biomarkers in cognitively normal people from the community. JAMA Neurol. 2016;73:85–92. 10.1001/jamaneurol.2015.3098 26595683 PMC4710552

[5] Bobinski M, Wegiel J, Wisniewski HM, et al Atrophy of hippocampal formation subdivisions correlates with stage and duration of Alzheimer disease. Dementia. 1995;6:205–210. 10.1159/000106948 7550600

[6] Kern A, Behl C. The unsolved relationship of brain aging and late-onset Alzheimer disease. Biochim Biophys Acta. 2009;1790:1124–1132. 10.1016/j.bbagen.2009.07.016 19632303

[7] Iqbal K, Alonso Adel C, Chen S, et al Tau pathology in Alzheimer disease and other tauopathies. Biochim Biophys Acta. 2005;1739:198–210. 10.1016/j.bbadis.2004.09.008 15615638

[8] Simic G, Spanic E, Langer Horvat L, Hof PR. Blood-brain barrier and innate immunity in the pathogenesis of Alzheimer's disease. Prog Mol Biol Transl Sci. 2019;168:99–145. 10.1016/bs.pmbts.2019.06.003 31699331

[9] Wisniewski KE, Wisniewski HM, Wen GY. Occurrence of neuropathological changes and dementia of Alzheimer's disease in Down's syndrome. Ann Neurol. 1985;17:278–282. 10.1002/ana.410170310 3158266

[10] Wang Y, Mandelkow E. Tau in physiology and pathology. Nat Rev Neurosci. 2016;17:22–35. 10.1038/nrn.2015.1 26631930

[11] Mufson EJ, Ikonomovic MD, Counts SE, et al Molecular and cellular pathophysiology of preclinical Alzheimer's disease. Behav Brain Res. 2016;311:54–69. 10.1016/j.bbr.2016.05.030 27185734 PMC4931948

[12] Iqbal K, Liu F, Gong CX. Tau and neurodegenerative disease: The story so far. Nat Rev Neurol. 2016;12:15–27. 10.1038/nrneurol.2015.225 26635213

[13] Braak H, Braak E. Frequency of stages of Alzheimer-related lesions in different age categories. Neurobiol Aging. 1997;18:351–357. 10.1016/s0197-4580(97)00056-0 9330961

[14] Thal DR, Del Tredici K, Braak H. Neurodegeneration in normal brain aging and disease. Sci Aging Knowledge Environ. 2004;2004:pe26. 10.1126/sageke.2004.23.pe26 15190177

[15] Karanth S, Nelson PT, Katsumata Y, et al Prevalence and clinical phenotype of quadruple misfolded proteins in older ddults. JAMA Neurol. 2020;77:1299–1307. 10.1001/jamaneurol.2020.1741 32568358 PMC7309572

[16] Latimer CS, Burke BT, Liachko NF, et al Resistance and resilience to Alzheimer's disease pathology are associated with reduced cortical pTau and absence of limbic-predominant age-related TDP-43 encephalopathy in a community-based cohort. Acta Neuropathol Commun. 2019;7:91. 10.1186/s40478-019-0743-1 31174609 PMC6556006

[17] Malek-Ahmadi M, Chen K, Perez SE, Mufson EJ. Cerebral amyloid angiopathy and neuritic plaque pathology correlate with cognitive decline in elderly non-demented individuals. J Alzheimers Dis. 2019;67:411–422. 10.3233/JAD-180765 30594928 PMC7717643

[18] NIA and the National Plan to Address Alzheimer's Disease . Updated 2021. https://www.nia.nih.gov/about/nia-and-national-plan-address-alzheimers-disease

[19] Braak H, Braak E. Neuropathological stageing of Alzheimer-related changes. Acta Neuropathol. 1991;82:239–259. 10.1007/bf00308809 1759558

[20] Brady DR, Mufson EJ. Alz-50 immunoreactive neuropil differentiates hippocampal complex subfields in Alzheimer's disease. J Comp Neurol. 1991;305:489–507. 10.1002/cne.903050311 2037717

[21] Hyman BT, Van Hoesen GW, Damasio AR, Barnes CL. Alzheimer's disease: Cell-specific pathology isolates the hippocampal formation. Science. 1984; 225:1168–1170. 10.1126/science.6474172 6474172

[22] Raichle ME, MacLeod AM, Snyder AZ, Powers WJ, Gusnard DA, Shulman GL. A default mode of brain function. Proc Natl Acad Sci U S A. 2001;98:676–682. 10.1073/pnas.98.2.676 11209064 PMC14647

[23] Leech R, Sharp DJ. The role of the posterior cingulate cortex in cognition and disease. Brain. 2014; 137(Pt 1):12–32. 10.1093/brain/awt162 23869106 PMC3891440

[24] Maddock RJ, Garrett AS, Buonocore MH. Remembering familiar people: The posterior cingulate cortex and autobiographical memory retrieval. Neuroscience. 2001;104:667–676. 10.1016/s0306-4522(01)00108-7 11440800

[25] Bergeron D, Beauregard JM, Soucy JP, et al Posterior cingulate cortex hypometabolism in non-amnestic variants of alzheimer's disease. J Alzheimers Dis. 2020;77:1569–1577. 10.3233/JAD-200567 32925054

[26] Minoshima S, Giordani B, Berent S, Frey KA, Foster NL, Kuhl DE. Metabolic reduction in the posterior cingulate cortex in very early Alzheimer's disease. Ann Neurol. 1997;42:85–94. 10.1002/ana.410420114 9225689

[27] Neth BJ, Graff-Radford J, Mielke MM, et al Relationship between risk factors and brain reserve in late middle age: Implications for cognitive aging. Front Aging Neurosci. 2019;11:355. 10.3389/fnagi.2019.00355 31998113 PMC6962238

[28] Zhou Y, Dougherty JH, Jr., Hubner KF, Bai B, Cannon RL, Hutson RK. Abnormal connectivity in the posterior cingulate and hippocampus in early Alzheimer's disease and mild cognitive impairment. Alzheimers Dement. 2008; 4:265–270. 10.1016/j.jalz.2008.04.006 18631977

[29] Fransson P, Marrelec G. The precuneus/posterior cingulate cortex plays a pivotal role in the default mode network: Evidence from a partial correlation network analysis. Neuroimage. 2008;42:1178–1184. 10.1016/j.neuroimage.2008.05.059 18598773

[30] Raichle ME . The brain's default mode network. Annu Rev Neurosci. 2015;38:433–447. 10.1146/annurev-neuro-071013-014030 25938726

[31] Sridharan D, Levitin DJ, Menon V. A critical role for the right fronto-insular cortex in switching between central-executive and default-mode networks. Proc Natl Acad Sci U S A. 2008;105:12569–12574. 10.1073/pnas.0800005105 18723676 PMC2527952

[32] Wang Z, Liang P, Jia X, et al The baseline and longitudinal changes of PCC connectivity in mild cognitive impairment: A combined structure and resting-state fMRI study. PLoS One. 2012;7:e36838. 10.1371/journal.pone.0036838 22629335 PMC3356348

[33] Lee PL, Chou KH, Chung CP, et al Posterior cingulate cortex network predicts alzheimer's disease progression. Front Aging Neurosci. 2020;12:608667. 10.3389/fnagi.2020.608667 33384594 PMC7770227

[34] Twine NA, Janitz K, Wilkins MR, Janitz M. Whole transcriptome sequencing reveals gene expression and splicing differences in brain regions affected by Alzheimer's disease. PLoS One. 2011;6:e16266. 10.1371/journal.pone.0016266 21283692 PMC3025006

[35] Bennett DA, Buchman AS, Boyle PA, Barnes LL, Wilson RS, Schneider JA. Religious orders study and Rush memory and aging project. J Alzheimers Dis. 2018;64(s1):S161–S189. 10.3233/JAD-179939 29865057 PMC6380522

[36] Mufson EJ, Ikonomovic MD, Styren SD, et al Preservation of brain nerve growth factor in mild cognitive impairment and Alzheimer disease. Arch Neurol. 2003;60:1143–1148. 10.1001/archneur.60.8.1143 12925373

[37] Bennett DA, Schneider JA, Bienias JL, Evans DA, Wilson RS. Mild cognitive impairment is related to Alzheimer disease pathology and cerebral infarctions. Neurology. 2005;64:834–841. 10.1212/01.WNL.0000152982.47274.9E 15753419

[38] Schneider JA, Arvanitakis Z, Leurgans SE, Bennett DA. The neuropathology of probable Alzheimer disease and mild cognitive impairment. Ann Neurol. 2009;66:200–208. 10.1002/ana.21706 19743450 PMC2812870

[39] Mufson EJ, Chen EY, Cochran EJ, Beckett LA, Bennett DA, Kordower JH. Entorhinal cortex beta-amyloid load in individuals with mild cognitive impairment. Exp Neurol. 1999;158:469–490. 10.1006/exnr.1999.7086 10415154

[40] Schneider JA, Aggarwal NT, Barnes L, Boyle P, Bennett DA. The neuropathology of older persons with and without dementia from community versus clinic cohorts. J Alzheimers Dis. 2009;18:691–701. 10.3233/JAD-2009-1227 19749406 PMC2853869

[41] Rall SC J, Weisgraber KH, Mahley RW. Human apolipoprotein E. The complete amino acid sequence. J Biol Chem. 1982;257:4171–4178. 7068630

[42] Folstein MF, Folstein SE, McHugh PR. Mini-mental state. A practical method for grading the cognitive state of patients for the clinician. J Psychiatr Res. 1975;12:189–198. 10.1016/0022-3956(75)90026-6 1202204

[43] Newell KL, Hyman BT, Growdon JH, Hedley-Whyte ET. Application of the National Institute on Aging (NIA)-Reagan Institute criteria for the neuropathological diagnosis of Alzheimer disease. J Neuropathol Exp Neurol. 1999;58:1147–1155. 10.1097/00005072-199911000-00004 10560657

[44] Mirra SS, Heyman A, McKeel D, et al The consortium to establish a registry for alzheimer's disease (CERAD). Part II. Standardization of the neuropathologic assessment of Alzheimer's disease. Neurology. 1991;41:479–486. 10.1212/wnl.41.4.479 2011243

[45] Jellinger KA, Bancher C. Neuropathology of Alzheimer's disease: A critical update. J Neural Transm Suppl. 1998;54:77–95. 10.1007/978-3-7091-7508-8_8 9850917

[46] Kelley CM, Perez SE, Mufson EJ. Tau pathology in the medial temporal lobe of athletes with chronic traumatic encephalopathy: A chronic effects of neurotrauma consortium study. Acta Neuropathol Commun. 2019;7:207. 10.1186/s40478-019-0861-9 31831066 PMC6909582

[47] Overk CR, Kelley CM, Mufson EJ. Brainstem Alzheimer's-like pathology in the triple transgenic mouse model of Alzheimer's disease. Neurobiol Dis. 2009;35:415–425. 10.1016/j.nbd.2009.06.004 19524671 PMC3085918

[48] Mai JrK, Paxinos G. The human nervous system. 3rd ed. Elsevier Academic Press; 2012:xi, 1415 p.

[49] Mai JrK, Assheuer J, Paxinos G. Atlas of the human brain. 2nd ed. Elsevier Academic Press; 2004:viii, 246 p.

[50] Jiang YJ, Cao SQ, Gao LB, et al Circular ribonucleic acid expression profile in mouse cortex after traumatic brain injury. J Neurotrauma. 2019;36:1018–1028. 10.1089/neu.2018.5647 30261810

[51] Sekar S, Geiger P, Cuyugan L, et al Identification of circular RNAs using RNA sequencing. J Vis Exp. 2019. 10.3791/59981 31789321

[52] Magoc T, Salzberg SL. FLASH: Fast length adjustment of short reads to improve genome assemblies. Bioinformatics. 2011;27:2957–2963. 10.1093/bioinformatics/btr507 21903629 PMC3198573

[53] Bolger AM, Lohse M, Usadel B. Trimmomatic: A flexible trimmer for Illumina sequence data. Bioinformatics. 2014;30:2114–2120. 10.1093/bioinformatics/btu170 24695404 PMC4103590

[54] Olney KC, Brotman SM, Andrews JP, Valverde-Vesling VA, Wilson MA. Reference genome and transcriptome informed by the sex chromosome complement of the sample increase ability to detect sex differences in gene expression from RNA-Seq data. Biol Sex Differ. 2020;11:42. 10.1186/s13293-020-00312-9 32693839 PMC7374973

[55] Ross MT, Grafham DV, Coffey AJ, et al The DNA sequence of the human X chromosome. Nature. 2005;434:325–337. 10.1038/nature03440 15772651 PMC2665286

[56] Pertea M, Pertea GM, Antonescu CM, Chang TC, Mendell JT, Salzberg SL. StringTie enables improved reconstruction of a transcriptome from RNA-seq reads. Nat Biotechnol. 2015;33:290–295. 10.1038/nbt.3122 25690850 PMC4643835

[57] Kozomara A, Birgaoanu M, Griffiths-Jones S. miRBase: From microRNA sequences to function. Nucleic Acids Res. 2019;47(D1):D155–D162. 10.1093/nar/gky1141 30423142 PMC6323917

[58] Dennis G J, Sherman BT, Hosack DA, et al DAVID: Database for annotation, visualization, and integrated discovery. Genome Biol. 2003;4:3.12734009

[59] Jiao X, Sherman BT, Huang da W, et al DAVID-WS: A stateful web service to facilitate gene/protein list analysis. Bioinformatics. 2012;28:1805–1806. 10.1093/bioinformatics/bts251 22543366 PMC3381967

[60] Benjamini Y, Hochberg Y. Controlling the false discovery rate: A practical and powerful approach to multiple testing. J R Stat Soc Ser B (Methodological). 1995;57:12.

[61] Benjamini Y, Drai D, Elmer G, Kafkafi N, Golani I. Controlling the false discovery rate in behavior genetics research. Behav Brain Res. 2001;125:279–284. 10.1016/s0166-4328(01)00297-2 11682119

[62] Hosack DA, Dennis G, Jr., Sherman BT, Lane HC, Lempicki RA. Identifying biological themes within lists of genes with EASE. Genome Biol. 2003; 4:R70. 10.1186/gb-2003-4-10-r70 14519205 PMC328459

[63] Mi H, Muruganujan A, Casagrande JT, Thomas PD. Large-scale gene function analysis with the PANTHER classification system. Nat Protoc. 2013;8:1551–1566. 10.1038/nprot.2013.092 23868073 PMC6519453

[64] Koopmans F, van Nierop P, Andres-Alonso M, et al SynGO: An evidence-based, expert-curated knowledge base for the synapse. Neuron. 2019;103:217–234.e4. 10.1016/j.neuron.2019.05.002 31171447 PMC6764089

[65] Agarwal V, Bell GW, Nam JW, Bartel DP. Predicting effective microRNA target sites in mammalian mRNAs. Elife 2015;4. 10.7554/eLife.05005 PMC4532895 26267216

[66] Papadopoulos GL, Reczko M, Simossis VA, Sethupathy P, Hatzigeorgiou AG. The database of experimentally supported targets: A functional update of TarBase. Nucleic Acids Res. 2009;37(Database issue):D155–D158. 10.1093/nar/gkn809 18957447 PMC2686456

[67] Vlachos IS, Zagganas K, Paraskevopoulou MD, et al DIANA-miRPath v3.0: Deciphering microRNA function with experimental support. Nucleic Acids Res. 2015;43(W1):W460–W466. 10.1093/nar/gkv403 25977294 PMC4489228

[68] Euskirchen GM, Rozowsky JS, Wei CL, et al Mapping of transcription factor binding regions in mammalian cells by ChIP: Comparison of array- and sequencing-based technologies. Genome Res. 2007;17:898–909. 10.1101/gr.5583007 17568005 PMC1891348

[69] Funk CC, Casella AM, Jung S, et al Atlas of transcription factor binding sites from ENCODE DNase hypersensitivity data across 27 tissue types. Cell Rep. 2020;32:108029. 10.1016/j.celrep.2020.108029 32814038 PMC7462736

[70] Hudson ME, Snyder M. High-throughput methods of regulatory element discovery. Biotechniques. 2006; 41:673, 675, 677 passim. 10.2144/000112322 17191608

[71] Love MI, Huber W, Anders S. Moderated estimation of fold change and dispersion for RNA-seq data with DESeq2. Genome Biol. 2014;15:550. 10.1186/s13059-014-0550-8 25516281 PMC4302049

[72] Mufson EJ, Counts SE, Ginsberg SD, et al Nerve growth factor pathobiology during the progression of alzheimer's disease. Front Neurosci. 2019;13:533. 10.3389/fnins.2019.00533 31312116 PMC6613497

[73] Mufson EJ, Bothwell M, Kordower JH. Loss of nerve growth factor receptor-containing neurons in Alzheimer's disease: A quantitative analysis across subregions of the basal forebrain. Exp Neurol. 1989;105:221–232. 10.1016/0014-4886(89)90124-6 2548888

[74] Cuello AC, Pentz R, Hall H. The Brain NGF Metabolic Pathway in Health and in Alzheimer's Pathology. Front Neurosci. 2019;13. 10.3389/fnins.2019.00062 PMC6379336 30809111

[75] Gauthier J, Joober R, Dubé MP, et al Autism spectrum disorders associated with X chromosome markers in French-Canadian males. Molecular Psychiatry. 2006:11. 16261168 10.1038/sj.mp.4001756

[76] Lee JH . Genetic evidence for cognitive reserve: Variations in memory and related cognitive functions. J Clin Exp Neuropsychol. 2003;25:594–613. 10.1076/jcen.25.5.594.14582 12815498

[77] Smith G, Rani A, Kumar A, Barter J, Foster TC. Hippocampal subregion transcriptomic profiles reflect strategy selection during cognitive aging. J Neurosci. 2020;40:4888–4899. 10.1523/JNEUROSCI.2944-19.2020 32376783 PMC7326352

[78] Yegla B, Foster TC. Operationally defining cognitive reserve genes. Neurobiol Aging. 2022;110:96–105. 10.1016/j.neurobiolaging.2021.08.015 34565615

[79] Bossers K, Wirz KT, Meerhoff GF, et al Concerted changes in transcripts in the prefrontal cortex precede neuropathology in Alzheimer's disease. Brain. 2010;133(Pt 12):3699–723. 10.1093/brain/awq258 20889584

[80] Katsel P, Li C, Haroutunian V. Gene expression alterations in the sphingolipid metabolism pathways during progression of dementia and Alzheimer's disease: A shift toward ceramide accumulation at the earliest recognizable stages of Alzheimer's disease? Neurochem Res. 2007;32:845–856. 10.1007/s11064-007-9297-x 17342407

[81] Cabeza R, Anderson ND, Locantore JK, McIntosh AR. Aging gracefully: Compensatory brain activity in high-performing older adults. Neuroimage. 2002;17:1394–1402. 10.1006/nimg.2002.1280 12414279

[82] Park DC, Welsh RC, Marshuetz C, et al Working memory for complex scenes: Age differences in frontal and hippocampal activations. J Cogn Neurosci. 2003;15:1122–1134. 10.1162/089892903322598094 14709231

[83] McQuail JA, Dunn AR, Stern Y, et al Cognitive reserve in model systems for mechanistic discovery: The Importance of longitudinal studies. Front Aging Neurosci. 2020;12:607685. 10.3389/fnagi.2020.607685 33551788 PMC7859530

[84] Mahady LJ, He B, Malek-Ahmadi M, Mufson EJ. Telomeric alterations in the default mode network during the progression of Alzheimer's disease: Selective vulnerability of the precuneus. Neuropathol Appl Neurobiol. 2021;47:428–440. 10.1111/nan.12672 33107640

[85] Walker JM, White CL, Farrell K, Crary JF, Richardson TE. Neocortical neurofibrillary degeneration in primary age-related tauopathy. J Neuropathol Exp Neurol. 2022;81:146–148. 10.1093/jnen/nlab113 34865093 PMC9115324

[86] Zammit AR, Yu L, Petyuk V, et al Cortical proteins and individual differences in cognitive resilience in older adults. Neurology. 2022;98:e1304–e1314. 10.1212/WNL.0000000000200017 35241503 PMC8967427

[87] Liang WS, Dunckley T, Beach TG, et al Gene expression profiles in anatomically and functionally distinct regions of the normal aged human brain. Physiol Genomics. 2007;28:311–322. 10.1152/physiolgenomics.00208.2006 17077275 PMC2259385

[88] Minoshima S, Frey KA, Cross DJ, Kuhl DE. Neurochemical imaging of dementias. Semin Nucl Med. 2004;34:70–82. 10.1053/j.semnuclmed.2003.09.008 14735460

[89] Lattanzi S, Cagnetti C, Foschi N, et al Adjunctive perampanel in older patients with epilepsy: A multicenter study of clinical practice. Drugs Aging. 2021;38:603–610. 10.1007/s40266-021-00865-3 34075567 PMC8266697

[90] Loerch PM, Lu T, Dakin KA, et al Evolution of the aging brain transcriptome and synaptic regulation. PLoS One. 2008;3:e3329. 10.1371/journal.pone.0003329 18830410 PMC2553198

[91] Fagerberg L, Hallstrom BM, Oksvold P, et al Analysis of the human tissue-specific expression by genome-wide integration of transcriptomics and antibody-based proteomics. Mol Cell Proteomics. 2014;13:397–406. 10.1074/mcp.M113.035600 24309898 PMC3916642

[92] Duff MO, Olson S, Wei X, et al Genome-wide identification of zero nucleotide recursive splicing in Drosophila. Nature. 2015;521:376–379. 10.1038/nature14475 25970244 PMC4529404

[93] Kosmidis S, Polyzos A, Harvey L, et al RbAp48 Protein is a critical component of GPR158/OCN signaling and ameliorates age-related memory loss. Cell Rep. 2018;25:959–973.e6. 10.1016/j.celrep.2018.09.077 30355501 PMC7725275

[94] Kanaumi T, Takashima S, Iwasaki H, Mitsudome A, Hirose S. Developmental changes in the expression of GABAA receptor alpha 1 and gamma 2 subunits in human temporal lobe, hippocampus and basal ganglia: An implication for consideration on age-related epilepsy. Epilepsy Res. 2006;71:47–53. 10.1016/j.eplepsyres.2006.05.019 16829043

[95] Song C, Orlandi C, Sutton LP, Martemyanov KA. The signaling proteins GPR158 and RGS7 modulate excitability of L2/3 pyramidal neurons and control A-type potassium channel in the prelimbic cortex. J Biol Chem. 2019;294:13145–13157. 10.1074/jbc.RA119.007533 31311860 PMC6721952

[96] Cao G, Meng G, Zhu L, et al Susceptibility to chronic immobilization stress-induced depressive-like behaviour in middle-aged female mice and accompanying changes in dopamine D1 and GABAA receptors in related brain regions. Behav Brain Funct. 2021;17:2. 10.1186/s12993-021-00175-z 33863350 PMC8052654

[97] Sutton LP, Orlandi C, Song C, et al Orphan receptor GPR158 controls stress-induced depression. Elife. 2018;7. 10.7554/eLife.33273 PMC5823542 29419376

[98] Altmann A, Cash DM, Bocchetta M, et al Analysis of brain atrophy and local gene expression in genetic frontotemporal dementia. Brain Commun. 2020;2. 10.1093/braincomms/fcaa122 PMC7667525 33210084

[99] Zhu M, Jia L, Li F, Jia J. Identification of KIAA0513 and other hub genes associated with alzheimer disease using weighted gene coexpression network analysis. Front Genet. 2020;11:981. 10.3389/fgene.2020.00981 33005179 PMC7483929

[100] Ostrovskaya OI, Orlandi C, Fajardo-Serrano A, Young SM Jr., Lujan R, Martemyanov KA. Inhibitory signaling to ion channels in hippocampal neurons is differentially regulated by alternative macromolecular complexes of RGS7. J Neurosci. 2018;38:10002–10015. 10.1523/JNEUROSCI.1378-18.2018 30315127 PMC6234300

[101] Khrimian L, Obri A, Ramos-Brossier M, et al Gpr158 mediates osteocalcin's regulation of cognition. J Exp Med. 2017;214:2859–2873. 10.1084/jem.20171320 28851741 PMC5626410

[102] Bell KF, Bennett DA, Cuello AC. Paradoxical upregulation of glutamatergic presynaptic boutons during mild cognitive impairment. J Neurosci. 2007;27:10810–10817. 10.1523/JNEUROSCI.3269-07.2007 17913914 PMC6672819

[103] Berchtold NC, Sabbagh MN, Beach TG, Kim RC, Cribbs DH, Cotman CW. Brain gene expression patterns differentiate mild cognitive impairment from normal aged and Alzheimer's disease. Neurobiol Aging. 2014;35:1961–1972. 10.1016/j.neurobiolaging.2014.03.031 24786631 PMC4067010

[104] Ceylan H . Integrated bioinformatics analysis to identify alternative therapeutic targets for Alzheimer's disease: Insights from a synaptic machinery perspective. J Mol Neurosci. 2021. 10.1007/s12031-021-01893-9 34414562

[105] Counts SE, Alldred MJ, Che S, Ginsberg SD, Mufson EJ. Synaptic gene dysregulation within hippocampal CA1 pyramidal neurons in mild cognitive impairment. Neuropharmacology. 2014;79:172–179. 10.1016/j.neuropharm.2013.10.018 24445080 PMC3951099

[106] Mangleburg CG, Wu T, Yalamanchili HK, et al Integrated analysis of the aging brain transcriptome and proteome in tauopathy. Mol Neurodegener. 2020;15:56. 10.1186/s13024-020-00405-4 32993812 PMC7526226

[107] Ikonomovic MD, Mufson EJ, Wuu J, Bennett DA, DeKosky ST. Reduction of choline acetyltransferase activity in primary visual cortex in mild to moderate Alzheimer's disease. Arch Neurol. 2005;62:425–430. 10.1001/archneur.62.3.425 15767507

[108] Fessel J . The paradox of opposite directions of gene expressions in MCI and AD suggests possible therapy to prevent progression of MCI to AD. Alzheimers Dement (N Y). 2020;6:e12003. 10.1002/trc2.12003 32258360 PMC7111579

[109] Weinberg RB, Mufson EJ, Counts SE. Evidence for a neuroprotective microRNA pathway in amnestic mild cognitive impairment. Front Neurosci. 2015;9:430. 10.3389/fnins.2015.00430 26594146 PMC4633499

[110] Weng FL, He L. Disrupted ubiquitin proteasome system underlying tau accumulation in Alzheimer's disease. Neurobiol Aging. 2021;99:79–85. 10.1016/j.neurobiolaging.2020.11.015 33422896

[111] Ginsberg SD, Hemby SE, Lee VM, Eberwine JH, Trojanowski JQ. Expression profile of transcripts in Alzheimer's disease tangle-bearing CA1 neurons. Ann Neurol. 2000;48:77–87. 10894219

[112] Tiernan CT, Ginsberg SD, Guillozet-Bongaarts AL, et al Protein homeostasis gene dysregulation in pretangle-bearing nucleus basalis neurons during the progression of Alzheimer's disease. Neurobiol Aging. 2016;42:80–90. 10.1016/j.neurobiolaging.2016.02.031 27143424 PMC4973891

[113] Keller JN, Gee J, Ding Q. The proteasome in brain aging. Ageing Res Rev. 2002;1:279–293. 10.1016/s1568-1637(01)00006-x 12039443

[114] Framework for Terms Used in Research of Reserve and Resilience . https://reserveandresilience.com/framework/

[115] Dai HJ, Wang CK, Chang NW, et al Statistical principle-based approach for recognizing and normalizing microRNAs described in scientific literature. Database (Oxford). 2019;2019. 10.1093/database/baz030 PMC6391575 30809637

[116] Fromm B, Keller A, Yang X, Friedlander MR, Peterson KJ, Griffiths-Jones S. Quo vadis microRNAs? Trends Genet. 2020;36:461–463. 10.1016/j.tig.2020.03.007 32544447

[117] Koepsell TD, Kurland BF, Harel O, Johnson EA, Zhou XH, Kukull WA. Education, cognitive function, and severity of neuropathology in Alzheimer disease. Neurology. 2008;70(19 Pt 2):1732–1739. 10.1212/01.wnl.0000284603.85621.aa 18160675

[118] Knopman DS, Jack CR Jr., Wiste HJ, et al 18F-fluorodeoxyglucose positron emission tomography, aging, and apolipoprotein E genotype in cognitively normal persons. Neurobiol Aging. 2014;35:2096–2106. 10.1016/j.neurobiolaging.2014.03.006 24702820 PMC4053507

[119] Wada M, Noda Y, Shinagawa S, et al Effect of education on Alzheimer's disease-related neuroimaging biomarkers in healthy controls, and participants with mild cognitive impairment and alzheimer's disease: A cross-sectional study. J Alzheimers Dis. 2018;63:861–869. 10.3233/JAD-171168 29689728

[120] Yu L, Lutz MW, Wilson RS, et al APOE ε4-TOMM40 ‘523 haplotypes and the risk of Alzheimer's disease in older Caucasian and African Americans. PLoS One. 2017. 10.1371/journal.pone.0180356 PMC5495438 28672022

[121] Siddarth P B, Merill D, et al Longer TOMM40 poly-T variants associated with higher FDDNP-PET medial temporal tau and amyloid binding. PLoS One. 2018;13. 10.1371/journal.pone.0208358 PMC6281258 30517207

[122] Linnertz C AL, Gottschalk W, et al The cis-regulatory effect of an Alzheimer's disease-associated poly-T locus on expression of TOMM40 and APOE genes. Alzehimers Dementia. 2014;10:541–551. 10.1016/j.jalz.2013.08.280 PMC4098029 24439168

[123] Hefferon TW GJ, Yurk CE, et al A variable dinucleotide repeat in the CFTR gene contributes to phenotype diversity by forming RNA secondary structures that alter splicing. Proc Natl Acad Sci. 2004;101:3504–3509. 14993601 10.1073/pnas.0400182101 PMC373492

[124] Arenaza-Urquijo EM, Vemuri P. Resistance vs resilience to Alzheimer disease: Clarifying terminology for preclinical studies. Neurology. 2018;90:695–703. 10.1212/WNL.0000000000005303 29592885 PMC5894932

[125] Stern Y . How can cognitive reserve promote cognitive and neurobehavioral health? Arch Clin Neuropsychol. 2021;36:1291–1295. 10.1093/arclin/acab049 34651645 PMC8517622

[126] Boros BD, Greathouse KM, Gentry EG, et al Dendritic spines provide cognitive resilience against Alzheimer's disease. Ann Neurol. 2017;82:602–614. 10.1002/ana.25049 28921611 PMC5744899

[127] Montine TJ, Cholerton BA, Corrada MM, et al Concepts for brain aging: Resistance, resilience, reserve, and compensation. Alzheimers Res Ther. 2019;11:22. 10.1186/s13195-019-0479-y 30857563 PMC6410486

[128] Johnson CB CE, Dammer EB, et al Large-scale deep multi-layer analysis of Alzheimer’s disease brain reveals strong proteomic disease-related changes not observed at the RNA level. Nature Neuroscience. 2022;25:213–225. 35115731 10.1038/s41593-021-00999-y PMC8825285

[129] Schott N, Krull K. Stability of lifestyle behavior - The answer to successful cognitive aging? A comparison of nuns, monks, master athletes and non-active older adults. Front Psychol. 2019;10:1347. 10.3389/fpsyg.2019.01347 31231291 PMC6567482

[130] McManus D . A phenomenological study of the lived experience of roman catholic sisters and successful aging. J Holist Nurs. 2020;38:350–361. 10.1177/0898010120913174 32193982

[131] Brooks-Wilson AR . Genetics of healthy aging and longevity. Hum Genet. 2013;132:1323–1338. 10.1007/s00439-013-1342-z 23925498 PMC3898394

[132] Keohane K, Balfe M. The Nun Study and Alzheimer's disease: Quality of vocation as a potential protective factor? Dementia (London). 2019;18:1651–1662. 10.1177/1471301217725186 28840756

[133] Kramer AF, Bherer L, Colcombe SJ, Dong W, Greenough WT. Environmental influences on cognitive and brain plasticity during aging. J Gerontol A Biol Sci Med Sci. 2004;59:M940–M957. 10.1093/gerona/59.9.m940 15472160

[134] Lim ASP, Gaiteri C, Yu L, et al Seasonal plasticity of cognition and related biological measures in adults with and without Alzheimer disease: Analysis of multiple cohorts. PLoS Med. 2018;15:e1002647. 10.1371/journal.pmed.1002647 30180184 PMC6122787

[135] Rohlfing T, Zahr NM, Sullivan EV, Pfefferbaum A. The SRI24 multichannel atlas of normal adult human brain structure. Hum Brain Mapp. 2010;31:798–819. 10.1002/hbm.20906 20017133 PMC2915788

