# Supplementary material for: Posterior cingulate cortex reveals an expression profile of resilience in cognitively intact elders
Source: Brain Commun. 2022 Jun 21;4(4):fcac162. doi: 10.1093/braincomms/fcac162 (PMC9263888; doi:10.1093/braincomms/fcac162)
Supplement: fcac162_Supplementary_Data [file fcac162_supplementary_data.docx]

**Supplementary Methods, Discussion, Tables, Figures and References**

## *Sequencing and processing*

An average of 68.9 million read pairs was obtained from the 26 subjects (range of 47.8 - 86.8 million) post-PCR duplicate removal. Prior to quality filtering, reads were merged by overlapped paired ends resulting in an average of 64 % of reads merged with an average length of 112 bases. Following trimming and quality filtering, 89 % of merged paired-end reads, 64 % of non-merged paired R1 and R2, 21 % unpaired R1, and 4 % unpaired R2 survived threshold cutoff. This resulted in an average of 61.4 million sequences and sequence pairs per subject with an average length of 95 bases available for mapping and alignment. Of these, an average of 60.3 million reads (98 %) per subject mapped to hg38; 30.4 million were counts for genes in our reference list and used for differential expression analysis.

## *Differential expression data*

Heatmaps and scatterplot visualizations using within-gene normalized counts^1^ revealed high variability within Braak stage I (n = 3), so the first comparison structure using a different grouping for Braak I was discarded (see Methods). Of non-shared genes, the p-value in the non-significant analysis was 0.09 in edgeR but was incalculable in DESeq2 due to more rigorous exclusion criteria resulting in discarding of genes with low counts (133 of the genes significant in edgeR were discarded from DESeq2 analysis). Of the 28,043 coding and noncoding genes annotated in the RefSeq feature file used for DESeq2 analysis, 811 genes were excluded from all comparisons due to low gene count, another 5,272 genes were removed from the analysis of Braak stage I/II with III, IV, or III/IV, and an additional 5,797 were excluded only from the comparison of Braak I/II with III. Encoding products related to transcription regulation that did not cluster in a functional enrichment pathway included histones, which influence nuclear chromatin structure.

**Supplementary Discussion (including a select response to issues raised by reviewers)**

## *Next generation sequencing*

We understand the concern for validation using a secondary technique such as qPCR. to confirm data obtained in large-scale transcriptomics studies, but whether this is necessary and/or provides an added value is not always clear. As stated in a recent commentary by T. Coenye, “Authors, reviewers and editors often struggle with this question . . .”^2^. Coenye points out that “It is not a priori known for which genes RNA-seq potentially yields non-concordant results in a particular study set up and as such it could be suggested to determine expression levels of all genes with qPCR or, alternatively, randomly select some genes for follow-up with qPCR. The former option is obviously not realistic in terms of cost and workload (and defeats the purpose of doing RNA-seq in the first place). The latter option could be an alternative, but how many genes need to be confirmed with another approach? As some genes are concordant and others are non-concordant, obtaining concordant results for a random selection of genes is no guarantee that other genes have been correctly identified as differentially expressed by RNA-seq and seems unlikely to provide much added value in most cases.”^2^ Based upon this discussion, we believe that validation of our RNA-Seq findings by using qRT-PCR is not required.

***Education***

We initially examined gene expression compared to demographics (e.g., age, education, among others) without consideration of Braak stage. As the results showing minimal and disjointed statistically significant findings, we decided to run differential expression with those variables individually but keep the treatment of further analyses relegated to differential expression as Braak-stage-specific. Based upon the difference between education medians (Table 1), we have addressed this issue of education level and Braak score in Supplementary Figure 1.

**Supplementary Table 1. Functional annotation clustering databases**

| DAVID ID | database source | ref. | Last updated (month.year) |
| --- | --- | --- | --- |
| BBID | Biological Biochemical Image Database | ^3^ | 03.2017 |
| BIOCARTA | BioCarta | ^4^ |  |
| BIOGRID_INTERACTION | Biological General Repository for Interaction Datasets | ^5^ | 02.2021 |
| COG_ONTOLOGY | Clusters of Orthologous Genes/ Clusters of Orthologous Groups of proteins | ^6^ |  |
| EC_NUMBER | IUBMB/IUPAC Biochemical Nomenclature Committee enzyme nomenclature | ^7^ |  |
| ENTREZ_GENE_SUMMARY | Entrez Molecular Sequence Database System | ^8^ | 03.2021 |
| GAD_DISEASE | Genetic Association Database | ^9^ | 08.2014 |
| GENERIF_SUMMARY | Gene Reference into Function (GeneRIF) |  | 03.2021 |
| GOTERM_BP_ | Gene Ontology Consortium (various), biological process | ^10^ | maintained |
| GOTERM_CC_ | ibid., cellular component | ^10^ |  |
| GOTERM_MF_ | ibid., molecular function | ^10^ |  |
| INTERPRO | InterPro | ^11^ | maintained |
| KEGG_PATHWAY | Kyoto Encyclopedia of Genes and Genomes | ^12^ |  |
| OMIM_DISEASE | Online Mendelian Inheritance in Man | ^13^ | maintained |
| PIR_SUMMARY/ PIR_SUPERFAMILY | Protein Information Resource | ^14^ |  |
| REACTOME_PATHWAY | reactome | ^15^ | maintained |
| SMART | Simple Modular Architecture Research Tool | ^16^ |  |
| SP_COMMENT | Swiss-Prot, manually reviewed Universal Protein Resource annotations | ^17^ |  |
| UCSC_TFBS | University of California, Santa Clara annotation file for transcription factor binding sites | ^18,19^ | 10.2010 |
| UP_KEYWORDS | UniProt hierarchy using a controlled vocabulary | ^20^ | 04.2018 |
| UP_SEQ_FEATURE | UniProt sequence annotation | ^20^ | 08.2020 |
| UP_TISSUE | UniProt tissue specificity and expression for mRNA and protein | ^20^ | 04.2020 |

**Supplementary Table 2.** **XY homologous genes and genes significantly differentially expressed in the posterior cingulate cortex in male (XY) and female (XX) aged non-cognitively impaired cases.**

| gene symbol | chr | male (XY)  TPM (PE, coverage)^1^ | female (XX)  TPM (PE, coverage) | DESeq2  FDR p | # of transcript variants  male, female | gene ID |
| --- | --- | --- | --- | --- | --- | --- |
| ARSD | X | 60 (100 %, 3) | 33 (100 %, 2) | < 1 E-6 | 2, 2 | 414 |
| CD24 ^2^ | 6 | 71 (100 %, 7) | 20 (93 %, 1) | < 0.05 | 3, 2 | 100133941 |
| DDX3X | X | 434 (100 %, 26) | 454 (100 %, 23) | ns | 3, 4 | 1654 |
| DDX3Y | Y | 16 (100 %, 4) | 25 (100 %, 1)* | < 1 E-6 | 4, 2 | 8653 |
| EIF1AX | X | 861 (100 %, 69) | 599 (93 %, 45) | ns | 1, 1 | 1964 |
| EIF1AY | Y | 124 (92 %, 6) | < 2 (21 %, < 1) | < 1 E-11 | 2, 1 | 9086 |
| HSFX1 | X | 6 (100 %, < 1) | 6 (100 %, < 1) | ns | 1, 1 | 100506164 |
| HSFY1 | Y | 3 (92 %, < 1) | 3 (100 %, < 1) | ns | 1, 1 | 86614 |
| HSFY1P1 | 22 | 10 (92 %, < 1) | < 2 (14 %, < 1) | < 1 E-3 | 1, 1 | 27437 |
| KDM5C | X | 87 (100 %, 5) | 93 (100 %, < 1) | < 0.05 | 3, 3 | 8242 |
| KDM5D | Y | 97 (100 %, 5) | 15 (100 %, 5) | < 1 E-14 | 2, 1 | 8284 |
| NLGN4X | X | 475 (100 %, 38) | 248 (100 %, 1) | < 1 E-2 | 1, 1 | 57502 |
| NLGN4Y | Y | 56 (100 %, 1) | 19 (100 %, 19) | < 1 E-3 | 5, 4 | 22829 |
| PCDH11X | X | 220 (100 %, 15) | 164 (100 %, < 1) | ns | 1, 2 | 27328 |
| PCDH11Y | Y | 47 (100 %, 3) | 40 (100 %, 9) | ns | 2, 2 | 83259 |
| PRKX | X | 123 (100 %, 10) | 84 (100 %, 7) | ns | 1, 1 | 5613 |
| PRKY | Y | 29 (100 %, 2) | 19 (100 %, 1) | ns | 1, 1 | 5616 |
| RPS4X | X | 3500 (100 %, 278) | 3275 (93 %, 255) | ns | 1, 1 | 6191 |
| RPS4Y1 | Y | 102 (92 %, 8) | < 2 (7 %, < 1) | < 1 E-16 | 1, 1 | 6192 |
| TBL1X | X | 15 (100 %, 1) | 12 (100 %, 1) | ns | 2, 2 | 6907 |
| TBL1Y | Y | 26 (100 %, 2) | 23 (100 %, 2) | ns | 1, 1 | 90665 |
| TMSB4X | X | 10,203 (100 %, 814) | 7590 (93 %, 579) | ns | 1, 1 | 7114 |
| TMSB4Y | Y | 38 (92 %, 3) | < 2 (14 %, < 1) | < 1 E-11 | 1, 1 | 9087 |
| TSIX | X | 69 (100 %, 4) | 547 (93 %, 43) | < 1 E-10 | 1, 1 | 9383 |
| TTTY10 | Y | 49 (92 %, 4) | < 2 (14 %, < 1) | < 1 E-12 | 1, 1 | 246119 |
| TTTY14 | Y | 471 (100 %, 11) | 3 (50 %, < 1) | < 1 E-12 | 6, 1 | 83869 |
| TTTY15 | Y | 64 (100 %, 5) | 7 (100 %, < 1) | < 1 E-13 | 1, 1 | 64595 |
| TXLNG | X | 650 (100 %, 29) | 525 (100 %, 23) | ns | 2, 2 | 55787 |
| TXLNGY | Y | 275 (100 %, 14) | < 2 (100 %, < 1) | < 1 E-16 | 1, 1 | 246126 |
| USP9X | X | 669 (100 %, 53) | 472 (93 %, 36) | ns | 1, 1 | 8239 |
| USP9Y | Y | 150 (100 %, 12) | < 2 (64 %, < 1) | < 1 E-11 | 1, 1 | 8287 |
| UTY | Y | 134 (100 %, 3) | 57 (100 %, 2) | < 0.001 | 14, 6 | 7404 |
| XIST | X | 221 (100 %, 13) | 1750 (93 %, 139) | < 1 E-9 | 1, 1 | 7503 |
| XKRX | X | 5 (75 %, < 1) | 3 (71 %, < 1) | ns | 1, 1 | 402415 |
| XKRY | Y | < 2 (50 %, < 1) | < 2 (21 %, < 1) | ns | 1, 1 | 9082 |
| ZFX | X | 43 (100 %, 3) | 49 (100 %, 3) | ns | 2, 2 | 7543 |
| ZFY | Y | 56 (100 %, 3) | < 2 (71 %, < 1) | < 1 E-8 | 2, 1 | 7544 |

^1^ Transcripts per million (TPM), percent of subjects expressed within (PE), and coverage based on reference-guided assembly (StringTie).

^2^ Differential expression statistical analysis in DESeq2 uses a normalized raw counts value extracted from a separate analysis than assembly.^1^

^3^ CD24 transcription was reported due to a pseudogene on chrY.

* Note due to similarity between X and Y chromosome segments there is cross mapping.^21,22^ All subjects, regardless of genotype, were mapped to somatic and X chromosomes and remaining unmapped reads were mapped to the Y chromosome without masking (see Methods). This minimizes XX humans mapping to Y but does not prevent it. Along similar lines, this analysis decreases what will map to Y in XY individuals. Although some prefer to mask the Y chromosome in XX humans, it does not prevent cross-mapping but it limits cross-mapping to one group. Evaluation of coverage, PE and transcript variants ais in elucidating sex chromosome expression differences.

**Supplementary Table 3. Genes of interest**

| gene symbol^1^ | assembly (StringTie 2.2.1) | | | |  | differential expression (DESeq2) | |  |
| --- | --- | --- | --- | --- | --- | --- | --- | --- |
|  | TPM^2^ | coverage | # of transcript variants (I/II,III,IV) | % subjects expressed |  | expression level CPM^3^ | compared to stage I/II^4^ | gene ID |
| ABCA7 | 52 | 6.0 | 1.0 (1,1,1) | 100 |  | 18 | ns | 10347 |
| ABCC9 | 24 | 0.9 | 1.0 (1,1,1) | 100 |  | 55 | ns | 10060 |
| ANK1 | 1118 | 10.2 | 3.0 (3,3,3) | 100 |  | 33 | ns | 286 |
| APOE | 1102 | 67.0 | 3.0 (3,3,3) | 100 |  | 161 | ns | 348 |
| APP | 7616 | 564.1 | 3.0 (3,3,3) | 100 |  | 557 | ns | 351 |
| ATP2B1 | 4785 | 105.5 | 6.0 (6,6,6) | 100 |  | 218 | ns | 490 |
| BDNF | 122 | 1.6 | 7.5 (7,8,8) | 100 |  | < 2 | ns | 627 |
| BIN1 | 13,501 | 116.3 | 6.0 (6,6,6) | 100 |  | 113 | ns | 274 |
| CCDC62 | 134 | 4.6 | 1.6 (1,2,2) | 100 |  | 6 | ns | 84660 |
| CD2AP | 100 | 9.2 | 1.0 (1,1,1) | 100 |  | 20 | ns | 23607 |
| CD33 | 26 | 2.0 | 1.8 (2,2,2) | 100 |  | < 2 | ns | 945 |
| CDKN2A | 84 | 0.8 | 3.6 (4,4,3) | 100 |  | < 2 | ns | 1029 |
| CDKN2B | 50 | 1.1 | 1.3 (1,1,1) | 100 |  | < 2 | ns | 1030 |
| CELF1 | 1612 | 32.0 | 9.0 (9,9,9) | 100 |  | 39 | ns | 10658 |
| CELSR2 | 949 | 83.3 | 1.0 (1,1,1) | 96 |  | 258 | ns | 1952 |
| CETP | 3 | 0.3 | 1.0 (1,1,1) | 96 |  | < 2 | ns | 1071 |
| CR1 | 12 | 0.6 | 1.8 (2,2,2) | 100 |  | 3 | ns | 1378 |
| ENC1 | 7680 | 202.0 | 2.0 (2,2,2) | 96 |  | 276 | ns | 8507 |
| EPHA1 | 10 | 0.3 | 1.0 (1,1,1) | 100 |  | < 2 | ns | 2041 |
| FADD | 136 | 7.4 | 1.0 (1,1,1) | 100 |  | 4 | ns | 8772 |
| FTO | 26 | 3.1 | 3.9 (4,4,4) | 100 |  | 528 | ns | 79068 |
| GAK | 4311 | 35.8 | 2.0 (2,2,2) | 96 |  | 56 | ns | 2580 |
| GRN | 217 | 27.7 | 1.0 (1,1,1) | 100 |  | 25 | ns | 2896 |
| HMGCR | 231 | 6.9 | 1.0 (1,1,1) | 100 |  | 72 | ns | 3156 |
| IGF2BP2 | 19 | 0.3 | 3.2 (4,3,3) | 100 |  | 9 | ↓IV ↓III/IV | 10644 |
| IRS1 | 487 | 13.5 | 1.0 (1,1,1) | 96 |  | 23 | ns | 3667 |
| KL | 112 | 1.0 | 1.0 (1,1,1) | 100 |  | < 2 | ns | 9365 |
| MAPT | 12 | 1.0 | 1.0 (1,1,1) | 100 |  | 732 | ns | 4137 |
| MED13 | 350 | 43.3 | 1.0 (1,1,1) | 100 |  | 121 | ns | 9969 |
| NMD3 | 693 | 19.7 | 1.0 (1,1,1) | 96 |  | 19 | ns | 51068 |
| PICALM | 4141 | 125.7 | 3.0 (3,3,3) | 100 |  | 136 | ns | 8301 |
| PTPRD | 5881 | 73.9 | 3.0 (3,3,3) | 100 |  | 220 | ns | 5789 |
| REST | 128 | 2.1 | 1.0 (1,1,1) | 100 |  | 26 | ↓III ↓III/IV | 5978 |
| RHBDF2 | 30 | 3.0 | 5.0 (5,5,5) | 100 |  | 43 | ns | 79651 |
| SORL1 | 1836 | 104.6 | 1.0 (1,1,1) | 96 |  | 461 | ns | 6653 |
| SREBF1 | 59 | 7.6 | 1.0 (1,1,1) | 100 |  | 82 | ns | 6720 |
| TARDBP | 393 | 36.3 | 1.0 (1,1,1) | 96 |  | 64 | ns | 23435 |
| TMEM106B | 671 | 14.3 | 2.0 (2,2,2) | 100 |  | 83 | ns | 54664 |
| TOMM40 | 38 | 4.0 | 2.0 (2,2,2) | 100 |  | 23 | ↑III | 10452 |
| TREM1 | 3 | 0.2 | 2.3 (3,2,2) | 100 |  | < 2 | ↓IV ↓III/IV | 54210 |
| TREM2 | 216 | 12.3 | 2.0 (2,2,2) | 100 |  | 3 | ns | 54209 |
| UNC5C | 1073 | 20.1 | 1.0 (1,1,1) | 96 |  | 23 | ns | 8633 |
| ZBED3-AS1 | 139 | 3.8 | 1.0 (1,1,1) | 100 |  | < 2 | ns | 728723 |
| ZCWPW1 | 253 | 4.4 | 1.8 (2,2,2) | 100 |  | 4 | ns | 55063 |

^1^ Based on Bennett, Buchman, et al., 2018^23^

^2^ transcripts per million calculated after reference-guided assembly in StringTie

^3^ counts per million calculated after DESeq2 normalized read counts^1^

^4^ ns, not significant

**Supplementary Table 4. Pathway analysis of significantly differentially expressed miRNAs using gene union merge**

| KEGG pathway name | KEGG pathway ID | miR-db (#) | genes (#) | FDR p |
| --- | --- | --- | --- | --- |
| cancer, pathways in | hsa05200 | 16 | 141 | < 0.0005 |
| PI3K-Akt signaling | hsa04151 | 16 | 115 | < 0.05 |
| MAPK signaling | hsa04010 | 15 | 91 | < 0.01 |
| Rap1 signaling | hsa04015 | 15 | 85 | < 0.00001 |
| endocytosis | hsa04144 | 15 | 76 | < 0.005 |
| cAMP signaling | hsa04024 | 15 | 73 | < 0.05 |
| cGMP-PKG signaling | hsa04022 | 15 | 64 | < 0.01 |
| FoxO signaling | hsa04068 | 15 | 61 | < 0.001 |
| viral carcinogenesis | hsa05203 | 15 | 60 | < 0.05 |
| protein processing in endoplasmic reticulum | hsa04141 | 15 | 57 | < 0.05 |
| hepatitis B | hsa05161 | 15 | 53 | < 0.05 |
| cancer, prostate | hsa05215 | 15 | 43 | < 0.00001 |
| oocyte meiosis | hsa04114 | 15 | 43 | < 0.05 |
| adherens junction | hsa04520 | 15 | 37 | < 0.00001 |
| focal adhesion | hsa04510 | 14 | 74 | < 0.05 |
| Ras signaling | hsa04014 | 14 | 73 | < 0.05 |
| Hippo signaling | hsa04390 | 14 | 65 | < 0.0000001 |
| oxytocin signaling | hsa04921 | 14 | 58 | < 0.05 |
| adrenergic signaling in cardiomyocytes | hsa04261 | 14 | 52 | < 0.05 |
| neurotrophin signaling | hsa04722 | 14 | 44 | < 0.05 |
| sphingolipid signaling | hsa04071 | 14 | 41 | < 0.05 |
| mRNA surveillance | hsa03015 | 14 | 35 | < 0.05 |
| cancer, pancreatic | hsa05212 | 14 | 31 | < 0.005 |
| proteoglycans in cancer | hsa05205 | 13 | 80 | < 0.000001 |
| actin cytoskeleton, regulation of | hsa04810 | 13 | 76 | < 0.05 |
| pluripotency of stem cells, regulation | hsa04550 | 13 | 61 | < 0.000001 |
| axon guidance | hsa04360 | 13 | 55 | < 0.000001 |
| insulin signaling | hsa04910 | 13 | 52 | < 0.05 |
| ubiquitin mediated proteolysis | hsa04120 | 13 | 52 | < 0.05 |
| estrogen signaling | hsa04915 | 13 | 42 | < 0.00001 |
| HIF-1 signaling | hsa04066 | 13 | 41 | < 0.05 |
| chronic myeloid leukemia | hsa05220 | 13 | 37 | < 0.0005 |
| progesterone-mediated oocyte maturation | hsa04914 | 13 | 34 | < 0.05 |
| phosphatidylinositol signaling | hsa04070 | 13 | 33 | < 0.005 |
| melanoma | hsa05218 | 13 | 32 | < 0.01 |
| bacterial invasion of epithelial cells | hsa05100 | 13 | 30 | < 0.05 |
| cancer, endometrial | hsa05213 | 13 | 24 | < 0.01 |
| Wnt signaling | hsa04310 | 12 | 56 | < 0.001 |
| cell cycle | hsa04110 | 12 | 48 | < 0.05 |
| choline metabolism in cancer | hsa05231 | 12 | 39 | < 0.05 |
| gap junction | hsa04540 | 12 | 35 | < 0.005 |
| renal cell carcinoma | hsa05211 | 12 | 34 | < 0.001 |
| long-term potentiation | hsa04720 | 12 | 33 | < 0.005 |
| cancer, colorectal | hsa05210 | 12 | 30 | < 0.005 |
| glioma | hsa05214 | 12 | 29 | < 0.001 |
| GABAergic synapse | hsa04727 | 12 | 28 | < 0.01 |
| cancer, non-small cell lung | hsa05223 | 12 | 23 | < 0.05 |
| circadian rhythm | hsa04710 | 12 | 16 | < 0.05 |
| glutamatergic synapse | hsa04724 | 11 | 44 | < 0.005 |
| ErbB signaling | hsa04012 | 11 | 42 | < 0.0005 |
| TGF-beta signaling | has04350 | 11 | 39 | < 0.000001 |
| acute myeloid leukemia | hsa05221 | 11 | 25 | < 0.05 |
| dorso-ventral axis formation | hsa04320 | 11 | 18 | < 0.0005 |
| nicotine addiction | hsa05033 | 10 | 18 | < 0.005 |
| thyroid hormone synthesis | hsa04918 | 9 | 25 | < 0.05 |
| mucin type O-glycan biosynthesis | hsa00512 | 9 | 11 | < 0.01 |
| Hedgehog signaling | hsa04340 | 8 | 23 | < 0.05 |
| glycosaminoglycan biosynthesis, CS/DS | hsa00532 | 8 | 9 | < 0.0005 |

CS, chondroitin sulfate; DS, dermatan sulfate; KEGG, Kyoto Encyclopedia of Genes and Genomes; miR-db, microRNA-database entities for significantly differentially expressed miRNA across Braak stages in three databases: TargetScan, microT-CDS, and Tarbase with DIANA

**Supplementary Table 5. Gene groupings negatively correlated with perceptual orientation**

| Cluster ID  Enrichment score^1^ | Pathway, gene ontology, or database name for group members^2^ (# of genes^3^) |
| --- | --- |
| LinOr.N.Cm1  1.43 [F/GO] | - single-organism cellular process/single-organism process/cellular process  - cell part/cell  - binding  - transcription factor binding site |
| LinOr.N.Cm2  1.40 [F/GO] | - cell adhesion/biological adhesion/T cell differentiation/single organismal cell-cell adhesion/single organism cell adhesion  - transcription factor binding site |
| LinOr.N.Cm3  1.21 [F/GO] | - extracellular exosome/extracellular vesicle/extracellular organelle/extracellular region/extracellular matrix  - structural molecule activity  - transcription factor binding site |
| LinOr.N.Cm4  1.21 [F/GO] | - multicellular organism growth/positive regulation of multicellular organismal process/cell morphogenesis involved in differentiation  - transcription factor binding site  - metal ion binding/cation binding/ion binding |
| LinOr.N.Cm5  1.13 [F/GO] | - cell adhesion/biological adhesion/cellular protein metabolic process/protein metabolic process/  - molecular transducer activity/receptor activity |
| PercOr.N.Cm5  1.10 [F/GO] | - Notch signaling pathway  - Hippo signaling pathway  - transcription factor activity, protein binding/protein binding  - nucleoplasm/nuclear lumen/catalytic complex/nuclear part/protein complex  - Type 2 Diabetes, edema, rosiglitazone |
| PercOr.N.Cm6  1.08 [F/GO] | - regulation of cell adhesion/positive regulation of cell adhesion/regulation of cell-cell adhesion/leukocyte aggregation/leukocyte cell-cell adhesion  - glycosaminoglycan binding  - extracellular matrix/extracellular region part |
| LinOr.N.Cm6  1.06 [F/GO] | - protein binding  - transcription factor binding site |
| PercOr.N.Cm8  0.90 [F/GO] | - transcription factor binding site  - Brain |
| PercOr.N.Dm1  0.87 [S/F] | - signal peptide/glycosylation site:N-linked (GlcNAc...)/disulfide bond  - Signal/Secreted/Glycoprotein/Disulfide bond |
| LinOr.N.Cm7  0.87 [F/GO] | - cell junction assembly/response to wounding/cell-cell junction organization/cell junction organization/circulatory system process  - cell surface/extracellular region/plasma membrane part/plasma membrane/cell periphery  - transcription factor binding site  - Brain |
| LinOr.N.Dm1  0.83 [S/F] | - glycosylation site:N-linked (GlcNAc...)/signal peptide/disulfide bond  - extracellular region/extracellular space  - Glycoprotein/Signal/Disulfide bond/Secreted |
| LinOr.N.Cm8  0.83 [F/GO] | - transcription factor binding site  - signal transduction/single organism signaling/signaling/cell communication/response to chemical |
| PercOr.N.Dm2  0.75 [S/F] | - positive regulation of transcription from RNA polymerase II promoter/regulation of transcription, DNA-templated  - Coiled coil/Nucleus/Transcription regulation/Transcription  - nucleoplasm/nucleus |
| LinOr.N.Cm9  0.75 [F/GO] | - transcription factor binding site  - cytoplasmic part/membrane-bounded organelle/intracellular organelle part/organelle/organelle part  - positive regulation of biological process/cellular response to stress/cellular component organization/cellular component organization or biogenesis/nitrogen compound metabolic process  - Tobacco Use Disorder  - Brain |
| LinOr.N.Cm10  0.72 [F/GO] | - regulation of epithelial cell differentiation/epithelial cell differentiation/tube development/mesenchyme development/positive regulation of multicellular organismal process  - transcription factor binding site  - hypertension  - nuclear part/intracellular organelle lumen/organelle lumen/membrane-enclosed lumen/nucleoplasm  - heterocyclic compound binding/organic cyclic compound binding |
| LinOr.N.Dm2  0.69 [S/F] | - cell surface/plasma membrane/integral component of membrane/membrane  - glycosylation site:N-linked (GlcNAc...)/topological domain: Extracellular/topological domain: Cytoplasmic/transmembrane region  - Glycoprotein/Cell membrane/Transmembrane helix/Transmembrane/Membrane |
| PercOr.N.Cm12  0.68 [F/GO] | - positive regulation of cell adhesion/apoptotic process/programmed cell death/cell death/  - extracellular matrix/extracellular region/extracellular region part/membrane-bounded vesicle/vesicle |
| PercOr.N.Cm13  0.68 [F/GO] | - cell development/response to steroid hormone/response to organic cyclic compound/single organism reproductive process/cellular response to chemical stimulus |
| LinOr.N.Cm11  0.60 [F/GO] | - macromolecule catabolic process/cellular catabolic process/organic substance catabolic process/cellular macromolecule catabolic process/catabolic process  - transcription factor binding site |
| LinOr.N.Cm12  0.49 [F/GO] | - transcription factor binding site  - endomembrane system |
| PercOr.N.Cm18  0.46 [F/GO] | - cellular component assembly/cellular component biogenesis/macromolecular complex subunit organization/macromolecular complex assembly/response to stress |
| LinOr.N.Cm13  0.37 [F/GO] | - transcription factor binding site  - cellular macromolecule localization/transport/establishment of localization/cellular localization/macromolecule localization  - cytosol |
| PercOr.N.Dm3  0.30 [S/F] | - external side of plasma membrane/plasma membrane/integral component of membrane  - topological domain: Extracellular/topological domain: Cytoplasmic/transmembrane region  - Transmembrane helix/Transmembrane/Membrane |
| PercOr.N.Cm27  0.23 [F/GO] | - regulation of cellular component organization/regulation of organelle organization/organelle organization  - carbohydrate derivative binding/purine ribonucleoside triphosphate binding/purine ribonucleoside binding/ribonucleoside binding/purine nucleoside binding |
| PercOr.N.Cm28  0.17 [F/GO] | - transcription factor binding site  - hydrolase activity/catalytic activity |

^1^[S/F] Structure and function enrichment analysis was performed using a combination of protein domain information, cellular localization, and some functional annotation. [F/GO] Function and gene ontological enrichment analysis was performed with a focus on functional pathways, protein interactions, gene ontology and tissue expression. ^2^ Since terms were combined for many database origins, it is possible to repeat terms or altered capitalization rules. The original format of each database has been preserved.

**Supplementary Table 6. Gene groupings positively correlated with perceptual orientation**

| Cluster ID  Enrichment score^1^ | Pathway, gene ontology, or database name for group members^2^ |
| --- | --- |
| PercOr.P.Cm4  1.93 [F/GO] | - axon/cation channel complex/axon part/neuron part/neuron projection  - nervous system development/regulation of membrane potential/inorganic cation transmembrane transport/action potential/multicellular organismal signaling  - cation channel activity/voltage-gated sodium channel activity/voltage-gated ion channel activity involved in regulation of postsynaptic membrane potential/voltage-gated ion channel activity/voltage-gated channel activity  - domain: The segment S4 is probably the voltage-sensor and is characterized by a series of positively charged amino acids at every third position.  - transcription factor binding site |
| PercOr.P.Dm1  1.90 [S/F] | - Ion transport domain/Voltage-dependent potassium channel, four helix bundle domain  - Voltage-gated channel/Ion channel/Sodium channel/Sodium transport/Ion transport  - dendrite/axon/integral component of membrane/integral component of plasma membrane/plasma membrane  - voltage-gated sodium channel activity/voltage-gated potassium channel activity  - regulation of postsynaptic membrane potential/sodium ion transmembrane transport/sodium ion transport/regulation of ion transmembrane transport/potassium ion transmembrane transport  - transmembrane region/glycosylation site:N-linked (GlcNAc...)/topological domain: Cytoplasmic |
| PercOr.P.Cm8  1.83 [F/GO] | - cellular process  - transcription factor binding site |
| PercOr.P.Cm9  1.71 [F/GO] | - positive regulation of biological process/regulation of cellular process/regulation of biological process/cellular process/single-organism cellular process  - cell part/cell  - protein binding/binding  - transcription factor binding site |
| PercOr.P.Cm11  1.62 [F/GO] | - myelination/axon ensheathment/ensheathment of neurons |
| PercOr.P.Cm12  1.61 [F/GO] | - neuron part/cell projection  - cellular component assembly/cellular component biogenesis/cellular component organization/cellular component organization or biogenesis/macromolecular complex subunit organization |
| PercOr.P.Cm17  1.09 [F/GO] | - regulation of nervous system development/regulation of cell development/regulation of neurogenesis/single-organism behavior/behavior |
| PercOr.P.Cm19  1.08 [F/GO] | - axon part/plasma membrane protein complex/synaptic membrane/axon terminus/membrane protein complex  - cellular component assembly/cellular component biogenesis/macromolecular complex assembly/protein complex assembly/protein complex biogenesis |
| PercOr.P.Cm20  1.07 [F/GO] | - cell projection membrane/plasma membrane region  - Amygdala |
| PercOr.P.Cm21  1.05 [F/GO] | - nervous system development/generation of neurons/neurogenesis/neuron development/neuron differentiation  - membrane region  - transcription factor binding site |
| PercOr.P.Cm22  0.95 [F/GO] | - ion transport/single-multicellular organism process  - transcription factor binding site  - plasma membrane part/membrane part/integral component of membrane/intrinsic component of membrane/membrane |
| PercOr.P.Cm23  0.95 [F/GO] | - glycerolipid biosynthetic process/phospholipid biosynthetic process/glycerophospholipid metabolic process/cellular lipid metabolic process/phosphatidylinositol biosynthetic process  - hydrolase activity, acting on ester bonds/hydrolase activity |
| PercOr.P.Cm25  0.88 [F/GO] | - positive regulation of cellular process/positive regulation of biological process/regulation of cell communication/regulation of signaling/single organism signaling  - transcription factor binding site  - cytosol |
| PercOr.P.Cm26  0.79 [F/GO] | - endoplasmic reticulum membrane/nuclear outer membrane-endoplasmic reticulum membrane network/endoplasmic reticulum part/endoplasmic reticulum/endomembrane system |
| PercOr.P.Dm3  0.78 [S/F] | - Synapse/Cell junction/Cell projection/Cell membrane  - cell junction |
| PercOr.P.Cm27  0.73 [F/GO] | - cytoplasmic part/cytoplasm/intracellular/intracellular part/organelle part |
| PercOr.P.Cm28  0.71 [F/GO] | - plasma membrane protein complex/membrane protein complex  - regulation of transport/nitrogen compound transport/regulation of localization/signal release/cell-cell signaling |
| PercOr.P.Dm4  0.63 [S/F] | - ATP binding  - Transferase/ATP-binding/Nucleotide-binding/Kinase  - nucleotide phosphate-binding region:ATP |
| PercOr.P.Cm31  0.55 [F/GO] | - adenyl ribonucleotide binding/adenyl nucleotide binding/small molecule binding/ATP binding/purine ribonucleotide binding  - organelle assembly/cellular response to organic substance/response to chemical  - transcription factor binding site |
| PercOr.P.Cm34  0.48 [F/GO] | - mitochondrion/mitochondrial part/mitochondrial inner membrane/organelle inner membrane/mitochondrial membrane  - oxidoreductase activity |
| PercOr.P.Cm36  0.45 [F/GO] | - plasma membrane region  - brain development/head development/animal organ development/central nervous system development |
| PercOr.P.Cm37  0.42 [F/GO] | - positive regulation of signaling/regulation of signal transduction/positive regulation of cell communication/regulation of response to stimulus/positive regulation of macromolecule metabolic process  - transcription factor binding site |
| PercOr.P.Cm38  0.41 [F/GO] | - mitochondrial part  - carbohydrate metabolic process/carboxylic acid metabolic process/oxoacid metabolic process/organic acid metabolic process/monocarboxylic acid metabolic process |
| PercOr.P.Cm41  0.37 [F/GO] | - whole membrane/bounding membrane of organelle/vacuolar membrane/vacuolar part/cytoplasmic, membrane-bounded vesicle |

^1^[S/F] Structure and function enrichment analysis was performed using a combination of protein domain information, cellular localization, and some functional annotation. [F/GO] Function and gene ontological enrichment analysis was performed with a focus on functional pathways, protein interactions, gene ontology, and tissue expression. ^2^Terms have been combined where database and number of genes matched, as this list pulls from many databases, it is possible to have a repeat in terms or altered capitalization rules. The original format of each database has been preserved.


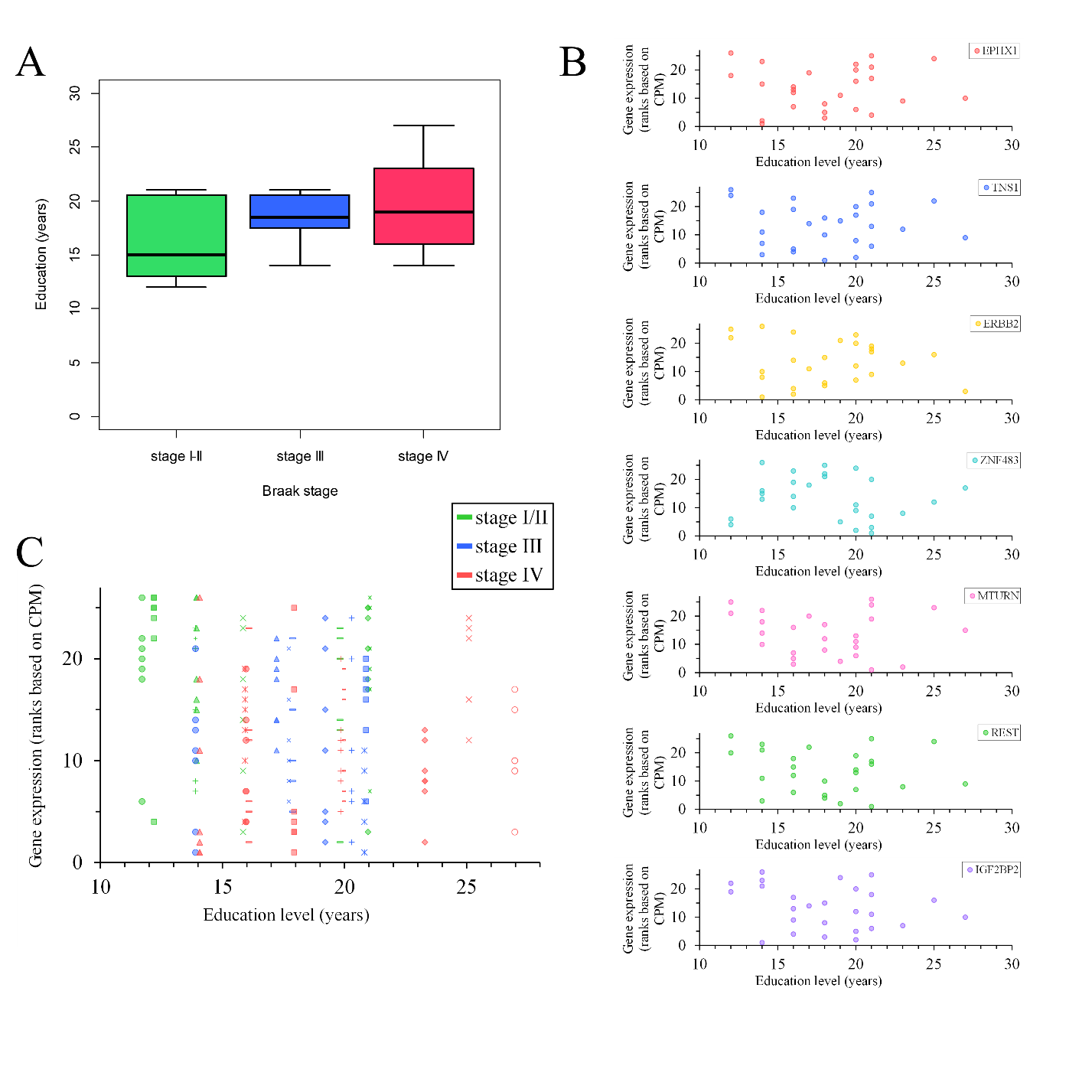


**Supplementary Figure 1**.  **Education level of cognitively normal aged adults relative to post-mortem Braak stages I/II (*n* = 8), III (*n* = 8), and IV (*n* = 10).** **A.** While median education level was lower in Braak stage I/II compared with stages III and IV, this was not statistically significant. **B.** Comparing select genes with education level shows no relationship with gene expression. Genes were chosen based on correlation with cognitive test score (*EPHX1*, *TNS1*, *ERBB2*, *ZNF483*) or high expression (*MTURN*), or RROS genes of interest (*REST*, *IGF2BP2*). For comparative purposes, the CPM from DESeq2 output was normalized using ranks for each gene. **C.** Expression of the same genes used in **B** is shown, colored by Braak Stage using a staggered scatterplot. In **A** and **C**, green represents Braak stage I/II, blue represents Braak stage III, and red represents Braak stage IV. In **C**, symbols represent different individuals with no association across stages for shared symbols.


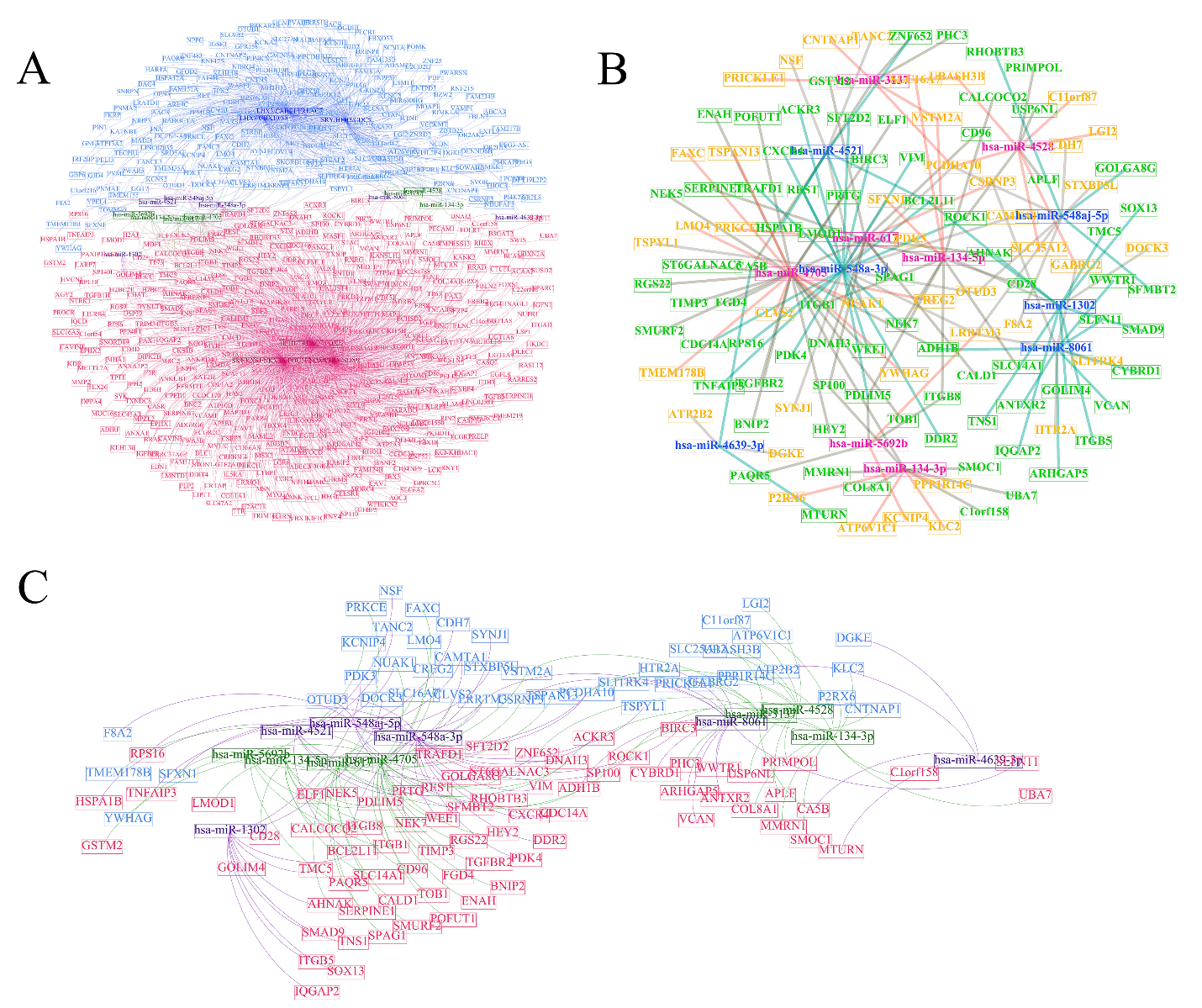


**Supplementary Figure 2. Network plots demonstrate directional changes in transcription factor binding sites (TFBSs), miRNA and associated mRNA from protein-coding genes.** **A.** TFBS and differentially expressed (DE) genes are shown in a network plot based on force-directed dispersion that shows a clear division between upregulated (blue, top) and downregulated (pink, bottom) DE genes with respect to Braak stage III (*n* = 8) or IV (*n* = 10) and stage I/II (*n* = 8). TFBS clusters are shown in darker colors of respective direction of change in the center of the mRNA with which they associate, representing hubs for this network. miRNA is shown in the middle of the two clusters as a consequence of the dispersion algorithm. Green labels and boxes represent miRNA upregulated in Braak stage IV or III/IV compared to stage I/II and purple represent those downregulated. **B.** Despite when miRNA and the mRNA they associate with are examined with respect to their direction of change, no clear association can be seen. The network does not demonstrate any spatial clustering (contrast with **A**) and individual direction of miRNA do not associate with direction of mRNA. Color scale is independent from **A** and **C**. With respect to Braak stage I/II: pink represents miRNA increased in Braak stage IV or III/IV; blue, miRNA decreased in Braak stage IV or III/IV; yellow, mRNA increased in Braak stage III, IV, or III/IV; and green, mRNA decreased in Braak stage III, IV, or III/IV. **C.** An enlargement of the miRNA and associated mRNA from **A** is shown. No dispersion or additional movement was applied. Colors are as in **A**.


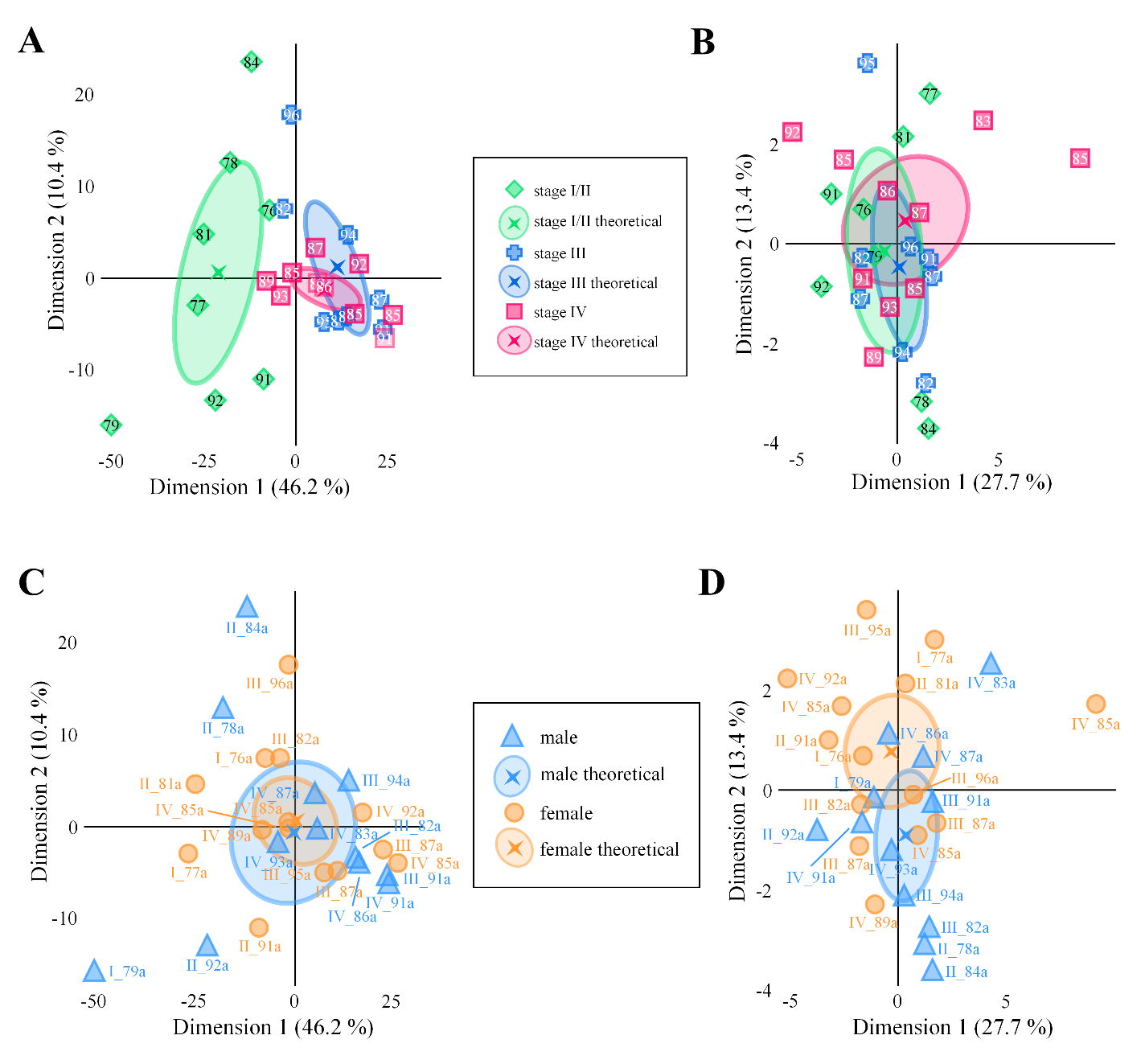


**Supplementary Figure 3**.  **Biplots derived from principal component analysis (PCA) using differentially expressed posterior cingulate cortex (PCC) genes (A, C) or cognitive test scores (B, D) from non-cognitively impaired elders (76-96 years at death).** **A.** Biplot generated with DESeq2 standard normalization protocol (calculated per gene) using the FactoMineR and factoextra package in R. A color palette was superimposed and data points formatted to show Braak stage (green diamond, stage I/II (*n* = 8); blue cross, stage III (*n* = 8); pink square, stage IV (*n* = 10)). Each data point represents a single individual and age is superimposed. Stars within the larger ellipses represent a theoretical average location for each group (confidence interval 0.95). **B.** Data was derived using the protocol applied in A but with the addition of cognitive test scores. Ellipses show distinctions between groups I/II and III or IV when PCA is calculated with gene expression, but not when determined using cognitive scores indicating a gene x Braak stage but not a cognitive x Braak interaction. **C, D.** In contrast, overlaying males (*n* = 12; blue triangle) and females (*n* = 14; orange circle) resulted in a separation for biplot coordinates by cognitive scores (**D**) but not gene expression levels. Since biplots **B** and **D** lack gene information in the model, cognitive scores were substituted for genes.

**Supplementary Material References**

1. Love MI, Huber W, Anders S. Moderated estimation of fold change and dispersion for RNA-seq data with DESeq2. *Genome Biol*. 2014;15(12):550. doi:10.1186/s13059-014-0550-8

2. Coenye T. Do results obtained with RNA-sequencing require independent verification? *Biofilm*. Dec 2021;3:100043. doi:10.1016/j.bioflm.2021.100043

3. Becker KG, White SL, Muller J, Engel J. BBID: the biological biochemical image database. *Bioinformatics*. Aug 2000;16(8):745-6. doi:10.1093/bioinformatics/16.8.745

4. Nishimura D. BioCarta. *Biotech Software & Internet Report*. 2001;2(3):117-120. doi:10.1089/152791601750294344

5. Stark C, Breitkreutz BJ, Reguly T, Boucher L, Breitkreutz A, Tyers M. BioGRID: a general repository for interaction datasets. *Nucleic Acids Res*. Jan 1 2006;34(Database issue):D535-9. doi:10.1093/nar/gkj109

6. Galperin MY, Wolf YI, Makarova KS, Vera Alvarez R, Landsman D, Koonin EV. COG database update: focus on microbial diversity, model organisms, and widespread pathogens. *Nucleic Acids Res*. Jan 8 2021;49(D1):D274-D281. doi:10.1093/nar/gkaa1018

7. McDonald AG, Boyce S, Tipton KF. ExplorEnz: the primary source of the IUBMB enzyme list. *Nucleic Acids Res*. Jan 2009;37(Database issue):D593-7. doi:10.1093/nar/gkn582

8. Murphy M, Brown G, Wallin C, et al. Gene Help: Integrated Access to Genes of Genomes in the Reference Sequence Collection. *Gene Help [Internet] Bethesda (MD): National Center for Biotechnology Information (US)*. 2021;

9. Becker KG, Barnes KC, Bright TJ, Wang SA. The genetic association database. *Nat Genet*. May 2004;36(5):431-2. doi:10.1038/ng0504-431

10. Kishore R, Arnaboldi V, Van Slyke CE, et al. Automated generation of gene summaries at the Alliance of Genome Resources. *Database (Oxford)*. Jan 1 2020;2020doi:10.1093/database/baaa037

11. Blum M, Chang HY, Chuguransky S, et al. The InterPro protein families and domains database: 20 years on. *Nucleic Acids Res*. Jan 8 2021;49(D1):D344-D354. doi:10.1093/nar/gkaa977

12. Kanehisa M, Goto S. KEGG: kyoto encyclopedia of genes and genomes. *Nucleic Acids Res*. Jan 1 2000;28(1):27-30. doi:10.1093/nar/28.1.27

13. Amberger JS, Bocchini CA, Scott AF, Hamosh A. OMIM.org: leveraging knowledge across phenotype-gene relationships. *Nucleic Acids Res*. Jan 8 2019;47(D1):D1038-D1043. doi:10.1093/nar/gky1151

14. Wu CH, Nikolskaya A, Huang H, et al. PIRSF: family classification system at the Protein Information Resource. *Nucleic Acids Res*. Jan 1 2004;32(Database issue):D112-4. doi:10.1093/nar/gkh097

15. Jassal B, Matthews L, Viteri G, et al. The reactome pathway knowledgebase. *Nucleic Acids Res*. Jan 8 2020;48(D1):D498-D503. doi:10.1093/nar/gkz1031

16. Letunic I, Khedkar S, Bork P. SMART: recent updates, new developments and status in 2020. *Nucleic Acids Res*. Jan 8 2021;49(D1):D458-D460. doi:10.1093/nar/gkaa937

17. Poux S, Arighi CN, Magrane M, et al. On expert curation and scalability: UniProtKB/Swiss-Prot as a case study. *Bioinformatics*. Nov 1 2017;33(21):3454-3460. doi:10.1093/bioinformatics/btx439

18. Consortium EP, Birney E, Stamatoyannopoulos JA, et al. Identification and analysis of functional elements in 1% of the human genome by the ENCODE pilot project. *Nature*. Jun 14 2007;447(7146):799-816. doi:10.1038/nature05874

19. Funk CC, Casella AM, Jung S, et al. Atlas of Transcription Factor Binding Sites from ENCODE DNase Hypersensitivity Data across 27 Tissue Types. *Cell Rep*. Aug 18 2020;32(7):108029. doi:10.1016/j.celrep.2020.108029

20. UniProt C. UniProt: the universal protein knowledgebase in 2021. *Nucleic Acids Res*. Jan 8 2021;49(D1):D480-D489. doi:10.1093/nar/gkaa1100

21. Olney KC, Brotman SM, Andrews JP, Valverde-Vesling VA, Wilson MA. Reference genome and transcriptome informed by the sex chromosome complement of the sample increase ability to detect sex differences in gene expression from RNA-Seq data. *Biol Sex Differ*. Jul 21 2020;11(1):42. doi:10.1186/s13293-020-00312-9

22. Ross MT, Grafham DV, Coffey AJ, et al. The DNA sequence of the human X chromosome. *Nature*. Mar 17 2005;434(7031):325-37. doi:10.1038/nature03440

23. Bennett DA, Buchman AS, Boyle PA, Barnes LL, Wilson RS, Schneider JA. Religious Orders Study and Rush Memory and Aging Project. *J Alzheimers Dis*. 2018;64(s1):S161-S189. doi:10.3233/JAD-179939
